# Distinct Expression Patterns and Clinical Associations of the *IRX* Gene Family Across Hormone-Sensitive Cancers

**DOI:** 10.3390/cancers18050726

**Published:** 2026-02-24

**Authors:** Amali Thennakoon, Achala Fernando, Jyotsna Batra

**Affiliations:** 1 School of Biomedical Sciences, Faculty of Health, Queensland University of Technology, Brisbane, QLD 4059, Australia; t2.thennakoon@hdr.qut.edu.au (A.T.); a.vitharanage@qut.edu.au (A.F.); 2 Translational Research Institute, The Centre for Genomics and Personalised Health, Queensland University of Technology, Brisbane, QLD 4102, Australia; 3 Faculty of Health Sciences and Medicine, Bond University, Gold Coast, QLD 4226, Australia

**Keywords:** *IRX* gene family, hormone-sensitive cancers, transcription factor, gene expression

## Abstract

**Background/Objectives:** The Iroquois (*IRX*) family of homeobox genes regulates critical developmental processes, and emerging evidence suggests that their dysregulation contributes to cancer progression, particularly in relation to cancer stemness. Although their expression appears to be influenced by hormonal regulation, their potential roles in hormone-sensitive cancers remain incompletely understood. **Methods:** In this study, we performed a comprehensive, exploratory analysis of all six Iroquois genes (*IRX1*–*IRX6*) across prostate, breast, ovarian, and endometrial cancers. Using large-scale publicly available transcriptomic datasets, we systematically examined *IRX* gene expression patterns and their associations with tumour progression, prognosis, hormone regulation, drug response, and cancer stemness. **Results:** *IRX3* and *IRX5* were consistently elevated in estrogen-dependent tumours and *IRX*2 and *IRX*4 were notably upregulated in prostate cancer. Despite evidence of estrogen receptor 1 (ESR1) and androgen receptor (AR) binding near several *IRX* promoters, estrogen treatment assays showed that ESR1 binding at promoters alone was insufficient to induce *IRX* transcription. Clinically, *IRX*2 expression was associated with favourable outcomes in breast, endometrial, and ovarian cancers and showed correlations with stemness-related signatures in prostate cancer. Similarly, *IRX*4 expression was associated with stemness features in prostate and endometrial cancers. In addition, *IRX*6 expression showed associations with reduced sensitivity to abiraterone, suggesting a potential link with therapeutic resistance in these tumours. **Conclusions:** Collectively, these findings highlight the context-dependent expression patterns and clinical associations of *IRX* genes across hormone-driven cancers. While largely correlative, this study provides a framework for future functional investigations and suggests that selected *IRX*s may have potential utility as biomarkers for disease stratification and treatment response in hormone-sensitive cancers.

## Simple Summary

Hormone-sensitive cancers such as prostate, breast, ovarian, and endometrial cancers are governed by hormone signals and often show differential responses to treatment. Understanding the genes involved in these cancers will help explain why some tumours behave more aggressively or become resistant to therapy. The Iroquois (*IRX*) gene family is known to play important roles during development, but their importance in hormone-sensitive cancers is not well understood. In this study, we analyzed large public datasets to examine how *IRX* genes are expressed in different hormone-sensitive cancers and how their expression correlates to disease progression, patient outcomes, cancer stem-like features, and treatment response. We found that *IRX* genes show distinct patterns depending on cancer type. These findings provide a useful resource for researchers and highlight that *IRX* genes may act as potential markers of tumour behaviour that warrant further investigation.

## 1. Introduction

Cancer remains a major global health challenge, ranking among the leading causes of premature mortality worldwide [1]. Hormone-sensitive cancers including prostate, breast, endometrial, and ovarian cancers represent a significant subset driven by steroid hormones such as estrogen, progesterone, and androgens [2]. Breast cancer is the most frequently diagnosed malignancy in women, while prostate cancer ranks second among men; both are leading causes of cancer-related deaths globally. Endometrial and ovarian cancers are also prevalent gynaecologic malignancies [3]. Despite advances in endocrine- related therapies, many patients with hormone-sensitive cancers develop therapy resistance and experience poor clinical outcomes [4]. Emerging evidence implicates cancer stem cells (CSCs) in resistance and tumour recurrence [5]. Identifying early diagnostic biomarkers, prognostic markers, and novel therapeutic targets is thus crucial for improving outcomes in hormone-sensitive cancers.

Cancer progression often hijacks developmental pathways [6]. Among developmental regulators, the Iroquois (*IRX*) gene family plays an important role in cell fate determination, tissue patterning and organogenesis during embryonic development via acting as transcriptional activators or repressors [7,8,9,10,11]. This family comprises six genes, *IRX1*, *IRX2*, *IRX3*, *IRX4*, *IRX5*, and *IRX6*, organized into two genomic clusters: Cluster A (*IRX1*, *IRX2*, *IRX4*) on chromosome 5 and Cluster B (*IRX3*, *IRX5*, *IRX6*) on chromosome 16 [8,9]. Although *IRX* genes are critical during development, their roles in cancer remain underexplored.

Emerging evidence shows that dysregulated *IRX* expression can contribute to oncogenesis as either tumour suppressors or oncogenes, varying by context [11,12,13,14,15,16,17,18,19,20,21]. Moreover, genome-wide association studies (GWAS) have identified cancer-associated genetic variants within the *5p15* locus, linking this region to lung, gastric, and prostate cancers [22,23,24,25]. Similarly, the *16q12* locus housing *IRX* Cluster B genes contains variants associated with retinoblastoma and breast cancer [26,27]. Beyond genetic variations, epigenetic modifications such as aberrant CpG island methylation have also been implicated in altering *IRX* gene expression in several malignancies [28].

Emerging evidence suggests that *IRX* genes may also be hormonally regulated, potentially influencing hormone-sensitive cancers. Our prior work demonstrated androgen regulation of alternative transcripts of *IRX4* and its involvement in prostate cancer progression [29]. In breast cancer, low *IRX2* shows a negative correlation with hormone receptor expression, and the reintroduction of *IRX2* into hormone-resistant cells reduces invasiveness and appears to be estrogen-regulated [30]. These observations raise the possibility that *IRX* genes serve as downstream effectors of hormone signalling pathways, potentially linking hormonal cues to processes such as cancer stemness and therapeutic resistance.

Expression profiling can help predict patient outcomes, identify therapeutic targets, and provide insights into cancer progression, including metastasis and advanced disease. With advances in next-generation sequencing, large-scale gene expression data are now available through public resources, enabling such investigations [31,32].

Given these considerations, we hypothesized that the *IRX* gene family may exhibit distinct, context-specific expression patterns across hormone-sensitive cancers and may be associated with clinical outcomes and treatment response. In this study, we utilized publicly available genomic and transcriptomic datasets to systematically characterize *IRX* gene expression in hormone-sensitive malignancies. We further examined their associations with tumour progression, patient prognosis, drug response, and cancer stemness-related features. Collectively, our analyses suggest that *IRX2*, *IRX3*, *IRX4*, and *IRX5* show cancer type-specific expression patterns and clinically relevant associations, with *IRX2* and *IRX4* warranting further investigation for their potential links to stemness-related pathways and therapeutic resistance in hormone-sensitive cancers.

## 2. Materials and Methods

### 2.1. Pan-Cancer Cell Line Transcriptome Atlas (PCTA) Datasets Analysis

The publicly available Pan-Cancer Cell Line Transcriptome Atlas (PCTA) database (https://pcatools.shinyapps.io/PCTA_app/ (accessed on 12 November 2024)) was used to assess the expression levels of each *IRX* gene across different hormone-sensitive cancer cell lines. Log2 (TPM + 1) expression data were visualized for each cancer type and *IRX* gene separately [33]. To ensure that gene expression analyses were not confounded by inactivating mutations, the mutation profiles of *IRX* family genes in the analyzed cancer cell lines were examined using the publicly available COSMIC (Catalogue of Somatic Mutations in Cancer) database. (The gene of interest and the cancer type were selected, and the generated graphs were directly downloaded.)

### 2.2. TNMplot Dataset Analysis

The TNMplot online gene expression array database [34] (https://tnmplot.com/analysis/ (accessed on 25 October 2024)) was used to analyze transcriptional differences in *IRX* family genes between primary tumour tissues and normal tissues in hormone-sensitive cancers. RNA-seq data for primary tumour tissue were obtained from The Cancer Genome Atlas (TCGA) through the TNMplot database. Normal tissue data comprised a combination of TCGA-adjacent normal tissues and healthy donor tissues from the GTEx database in the TNMplot database. Expression values were normalized in the TNMplot database using DESeq normalization followed by secondary scaling to reduce batch effects. Normalized expression values for all *IRX* genes in breast, ovarian, endometrial, and prostate cancers and normal tissues were downloaded. Boxplots illustrating differential expression between tumour and normal tissues were generated using GraphPad Prism 10.4.1. One-way ANOVA was applied to assess statistical significance. Additionally, a heatmap was generated using Metaboanalyst 6.0 [35] (https://www.metaboanalyst.ca/ (accessed on 15 November 2024)) to provide better visualization of the differential expression of each gene across the different cancers. (Data were generated using the section under gene expression comparison, TN-plot: compare tumour and normal section. RNA-seq data were downloaded for all six genes for four cancers. Normal samples of non-cancerous patients and additional pediatric tissue options were used as normal tissue data for the analysis.)

### 2.3. UALCAN Analysis

The UALCAN database [36] (http://ualcan.path.uab.edu (accessed on 24 November 2024)), which includes TCGA primary tumour tissues and normal tissue RNA seq data (TPM), was used to analyze the mRNA levels of *IRX* family genes in relation to tumour progression from Stage I to IV. (Accessed on 24 November 2024. TCGA gene expression data were selected for four cancers separately for each gene. Graphs showing gene expression for individual cancer stages were directly downloaded.)

### 2.4. GEPIA, Kaplan–Meier and Prognoscan Database Analysis

To assess the prognostic value of each *IRX* gene, we analyzed data from the GEPIA database (http://gepia2.cancer-pku.cn/#index (accessed on 5 March 2025)) [37] and the Kaplan–Meier Plotter dataset (https://kmplot.com/analysis/ (accessed on 3 March 2025)) and PrognoScan [38]. The GEPIA database, which provides access to TCGA data, was used to examine the correlation of each *IRX* gene with overall survival (OS) and disease-free survival (DFS) across all four cancers. Patient groups were classified based on the highest and lowest quartiles of *IRX* expression. In the Kaplan–Meier database which contains RNA seq_PANCANCER data, the optimal cutoff for defining expression groups was determined automatically and analyses were performed to investigate OS and relapse-free survival (RFS) in breast, ovarian and endometrial cancer datasets. The analyses included the calculation of hazard ratios (HRs) with corresponding 95% confidence intervals (CIs) and log-rank *p*-values. Differences in data processing pipelines, internal normalization methods, patient filtering criteria, and cutoff selection strategies may be cause for variations in statistical significance between platforms. PrognoScan (http://www.prognoscan.org/ (accessed on 4 April 2025)) is a database containing a large collection of publicly available cancer microarray datasets with clinical annotations. In PrognoScan, survival groups were classified using the portal’s default minimum *p*-value approach, which systematically evaluates multiple gene expression thresholds and selects the cutoff yielding the most statistically significant survival difference for each dataset. PrognoScan incorporates internal statistical adjustments associated with its optimal cut-point algorithm to mitigate multiple testing effects. All survival statistics were obtained using the default analytical settings of the respective portals, and no additional independent multiple testing corrections were applied beyond those implemented within the databases. (Kaplan–Meier Plotter: RNA seq data for pan-cancer were selected. GEPIA: Gene name, method and group cutoff were added as described above for respective cancer types. Prognoscan: Survival data for each gene were checked and data were selected only for hormone-sensitive cancers.)

### 2.5. Cistrome Data Browser Analysis

In this study, we used the Cistrome data browser [39] (http://cistrome.org/db/#/ (accessed on 25 May 2024)) to identify the Chip-seq peaks corresponding to the androgen receptor (AR), estrogen receptor 1 (ESR1), estrogen receptor 2 (ESR2), and progesterone receptor (PGR) in cell lines derived from breast, ovarian, endometrial, and prostate cancers. Only samples with the highest quality control (visualized as six green dots) were included. The UCSC Genome Browser [40] (https://genome.ucsc.edu/ (accessed on 27 May 2024)) was used to visualize these peaks on the genome. (Chip-seq data for homo sapiens were selected. All possible biological sources under hormone-sensitive cancers were examined. ESR1, AR, ESR2 and PGR were selected as factors. Only the samples with highest quality control were selected for further analysis.)

### 2.6. Association of IRX Gene Expression with Drug Sensitivity

Drug sensitivity Z-score values and gene expression (log2 (FPKM + 1)) data for each cell line were downloaded from the CellMiner database (https://discover.nci.nih.gov/cellminer/ (accessed on 30 September 2024)) [41]. In this study, drug sensitivity scores for FDA-approved drugs for breast, ovarian, and prostate cancer were selected. Correlation coefficients and adjusted *p*-values were calculated to assess the relationship between each *IRX* gene’s expression and drug sensitivity in hormone-sensitive cancer cell lines using the “Hmisc” package in R (4.4.1). Spearman’s correlation was used to calculate the correlation coefficients. The Benjamini–Hochberg (BH) method was used to obtain the adjusted *p*-values for multiple testing. Additionally, scatter plots were generated using the “corrplot” package to visualize the significant correlations. (Under cell line signatures, drug sensitivity Z-score values and RNA-seq gene expression values only for *IRX1-6* were downloaded for the analysis.)

### 2.7. Association of IRX Gene Expression with Cancer Stemness

RNA stemness scores, which are based on the expression of stemness-related genes, and *IRX* gene expression (log2(RNAseq-batch effect normalized gene expression + 1)) of breast, ovarian, endometrial and prostate cancer patients from the TCGA pan-cancer (PANCAN) dataset were downloaded from the UCSC Xena database (https://xena.ucsc.edu/ (accessed on 15 August 2024)) [42]. Correlation analysis was performed using the R platform (R 4.4.1), and Pearson’s correlation was used to calculate the correlation coefficients. Heatmaps were generated using the “ggplot” package for visualization. (TCGA PANCAN gene expression data were downloaded under the stemness score–RNA expression-based category.)

### 2.8. Cell Culture

A panel of cell lines representing prostate cancer (LNCaP (passage number(P)32, C42B(P33)), breast cancer (MCF-7(P5), MDA-MB-231(P15)), ovarian cancer (SKOV3 (P25), CAOV3 (P24)) and endometrial cancer (Ishikawa (P30), Hec-1A (P17)) cell lines were purchased from the American Type Culture Collection (ATCC, Manassas, VA, USA). LNCaP, C42B, SKOV3 and Hec-1A cell lines were grown in RPMI1640 (1X) with no phenol red (Life Technologies, Grand Island, NY, USA) supplemented with either 5% or 10% fetal bovine serum (FBS, Life Technologies, Thornton, Australia). MCF-7, MDA-MB-231, CAOV3 and Ishikawa cell lines were grown in DMEM (1X) with phenol red (Life Technologies, Grand Island, NY, USA) supplemented with 10% FBS. Cells were passaged at 70–80% confluence. The detachment of all cell lines was done with Trypsin/EDTA Solution (TE, Life Technologies, Grand Island, NY, USA). The cell lines were authenticated by short tandem repeat (STR) profiling and tested negative for Mycoplasma. The cells were maintained at 37 °C in a 5% CO_2_ humidified incubator (Panasonic, Oizumi-Machi, Japan).

### 2.9. Androgen and Estrogen Treatment

MCF-7 and Ishikawa cell lines were grown in 6-well plates using DMEM with no phenol red (Life Technologies, Grand Island, NY, USA) supplemented with 10% fetal bovine serum (FBS, Life Technologies, Thornton, Australia) and were incubated at 37 °C for 48 h. The medium was replaced with a hormone-depleted culture medium (DMEM) containing 5% charcoal-stripped serum (CSS, Sigma-Aldrich, Castle Hill, Australia) for 48 h. Next, one set of cells was supplemented with estrogen: 17β-Estradiol (E2) (10 nmol/L, Sigma-Aldrich, Castle Hill, Australia). At the same time another set of cells was treated with estrogen inhibitor, 4-Hydroxytamoxifen (10 µmol/L, 4-OHT, Merck Life Science, Truganina, Australia), along with E2 (10 nmol/L). Ethanol (EtOH) (0.02%, HPLC grade, Sigma-Aldrich, Castle Hills, Australia) was the control and incubated at 37 °C for an additional 48 h, as described previously [29].

### 2.10. RNA Isolation from Cell Lines

Total RNA was extracted from cancer cells (C42B, LNCaP, CAOV3, SKOV3, MDA-MB-231, MCF-7, Ishikawa, Hec-1A) using the Isolate II RNA Mini Kit (Bioline, London, UK). DNase digestion (Bioline, London, UK) were performed on the column during the extraction process. RNA concentration and purity was measured at 260/280 absorbance using a NanoDropTM1000 (Thermo Scientific, BiolaB, Scoresby, Australia).

### 2.11. cDNA Synthesis and Quantitative Real-Time Polymerase Chain Reaction (qRT-PCR)

For this step, 1 μg of isolated RNA was reverse transcribed to cDNA using the SensiFastTM cDNA synthesis kit (Bioline, GmbH, Luckenwalde, Germany). The cDNA was diluted to 100 μL before using it as a template for qRT-PCR assay. The primers for qRT-PCR were designed using the NCBI tool Primer-BLAST–NCBI–NIH software (Primer3 version 2.5.0) to cover all the splice variants of the respective gene. All the primer sequences are given in Supplementary Table S1. qRT-PCR was performed using the ViiA7 Real-Time PCR system (Applied Biosystems, Foster City, CA, USA). Each reaction contained 1X final concentration of SYBR Green PCR Master Mix (2X) (Applied Biosystems, Foster City, CA, USA), 50 nmol/L forward and reverse primer, 2 μL of diluted cDNA (1:5), and nuclease-free water at a final volume of 8 μL. The cycling parameters were 95 °C for 10 min, 40 cycles of 95 °C for 15 s, and 60 °C for 1 min followed by a dissociation step. All the CT values were normalized to the expression of the housekeeping gene RPL32 (ΔCT). Relative expression compared to control was determined using the comparative CT (ΔΔCT) method. Relative expression values (2^-ΔΔCt) were log2-transformed prior to visualization to enable scale comparability with RNA-seq datasets.

## 3. Results

### 3.1. Expression of IRX Genes in Hormone-Sensitive Cancer Cell Lines

**Figure 1:**
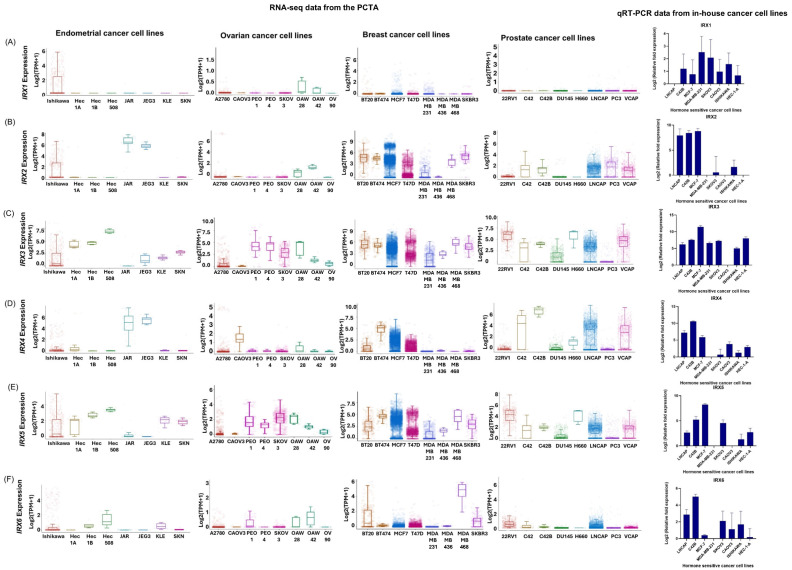
*IRX* gene expression in hormone-sensitive cancer cell lines. (**A**) Expression of *IRX*1 in endometrial, ovarian, breast and prostate cancer cell lines from PCTA database and from qRT-PCR. (**B**) Expression of *IRX*2 in endometrial, ovarian, breast and prostate cancer cell lines from PCTA database and from qRT-PCR. (**C**) Expression of *IRX*3 in endometrial, ovarian, breast and prostate cancer cell lines from PCTA database and from qRT-PCR. (**D**) Expression of *IRX*4 in endometrial, ovarian, breast and prostate cancer cell lines from PCTA database and from qRT-PCR. (**E**) Expression of *IRX*5 in endometrial, ovarian, breast and prostate cancer cell lines from PCTA database and from qRT-PCR. (**F**) Expression of *IRX*6 in endometrial, ovarian, breast and prostate cancer cell lines from PCTA database and from qRT-PCR (PCTA [1], Log2(TPM + 1)). The quantitative expression of *IRX* genes in panel of prostate, breast, ovarian and endometrial cancer cell lines (LNCaP, C42B, SKOV3, CAOV3, Ishikawa, HEC-1A, MDA-MB-231, MCF-7). RPL32 was used as the endogenous housekeeping control. The relative fold expression was determined using the ΔΔCT method with respect to the lowest expression in cell lines for each *IRX*. (*IRX*1 with respect to LNCAP, *IRX*2 with respect to HEC-1A, *IRX*3 with respect to CAOV3, *IRX*4, *IRX*5 and *IRX*6 with respect to MDA-MB-231) (n = 3 biological and n = 3 technical replicates, mean ± SD).

To investigate the differential expression of *IRX* gene family members, we initially performed an in silico analysis using RNA-seq data from the PCTA, covering breast, prostate, endometrial, and ovarian cancer cell lines. To validate these findings, qRT-PCR was conducted on a panel of in-house cancer cell lines representing the same cancer types. First, mutation data were analyzed in all cell lines. The review of mutation data from the COSMIC database indicated no recurrent inactivating mutations in the majority of the analyzed cell lines; where data were available, most reported variants were coding-silent substitutions. *IRX1* exhibited generally low expression across most cell lines in the RNA-seq dataset, a trend that was similarly observed in the in-house qRT-PCR analysis (Figure 1A). *IRX2* demonstrated comparatively higher expression in several breast and prostate cell lines in both datasets, although variability was noted among specific lines (Figure 1B). *IRX3* showed elevated expression across multiple cancer types in both RNA-seq and qRT-PCR analyses, except for CAOV3, where expression remained low (Figure 1C). *IRX4* expression was more prominent in breast and prostate cell lines, with moderate levels detected in selected endometrial and ovarian lines in both datasets (Figure 1D). *IRX5* displayed relatively high expression across several cell lines representing all four cancers in both analyses (Figure 1E). In contrast, *IRX6* expression was generally low across most cancers in RNA-seq data, whereas qRT-PCR revealed comparatively higher expression in prostate cancer cell lines (LNCaP and C42B) (Figure 1F). These consistent, cancer type-specific patterns suggest potential associations between *IRX* gene expression and oncogenic processes in hormone-sensitive cancers. The characteristics of the cell lines used in this study are included in Supplementary Table S2.

### 3.2. Differential Expression of IRXs in Hormone-Sensitive Cancer Patients

**Figure 2:**
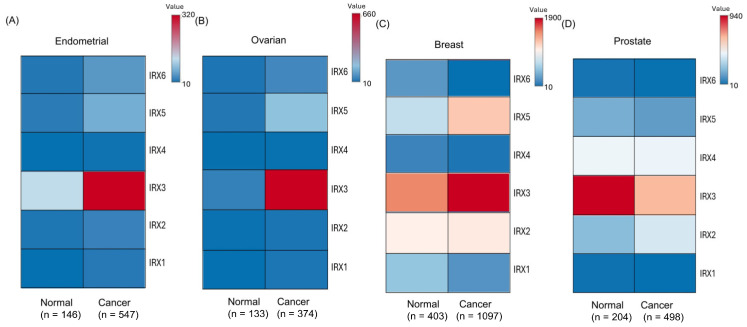
Differential expression of *IRX* family genes in hormone-sensitive cancer. (**A**) Expression of *IRX* family members in endometrial cancer tissues (n = 547) compared to normal endometrial non-cancerous tissues (n = 146). (**B**) Expression of *IRX* family members in ovarian serous cystadenocarcinoma tissues (n = 374) compared to normal ovarian tissues from non-cancerous patients (n = 133). (**C**) Expression of *IRX* family members in breast cancer tissues (n = 263) compared to normal breast tissues (n = 1562). (**D**) Expression of *IRX* family members in Prostate adenocarcinoma tissues (n = 499) compared to normal prostate tissues from non-cancerous patients (n = 203) (TNMplot, DESeq2 normalized RNA-seq expression data from TCGA and GTEx combined).

To investigate the clinical relevance of *IRX* family members, we analyzed differential expression across four hormone-sensitive cancer types using a combined dataset of RNA-seq datasets from the TCGA and GTEx datasets including primary hormone-sensitive cancer tissues and healthy tissues. Expression heatmaps revealed that *IRX*3 exhibited the highest expression among all *IRX* genes (Figure 2A–D). *IRX*3 was significantly upregulated in tumour tissues compared to normal tissues in endometrial, ovarian, and breast cancers, followed by *IRX*5 (Figure 2A–C). Conversely, *IRX*2 was notably upregulated in prostate cancer (Figure 2D). Detailed analyses using the same dataset confirmed these trends, showing significant upregulation of *IRX*3 and *IRX*5 in endometrial, breast, and ovarian cancers (Supplementary Figure S1). In addition, *IRX*4 was upregulated in endometrial cancer but generally expressed at low levels (Supplementary Figure S1). *IRX*1, *IRX*4, and *IRX*6 were upregulated in ovarian cancer at low levels and downregulated in breast cancer (Supplementary Figure S1). *IRX*2 was downregulated in breast cancer (Supplementary Figure S1). Although heatmaps indicated *IRX*2 as the most prominently upregulated gene in prostate cancer, individual analyses revealed significant *IRX*4 upregulation (Supplementary Figure S1). In contrast, *IRX*1, *IRX*3, *IRX*5, and *IRX*6 were significantly downregulated in prostate cancer (Supplementary Figure S1). Other *IRX* genes did not show significant differential expression across the cancer types analyzed. Collectively, these results demonstrate distinct, cancer type-specific expression patterns of *IRX* genes across hormone-sensitive malignancies.

### 3.3. Association of IRX Family Members with Progression of Hormone-Sensitive Cancers

**Figure 3:**
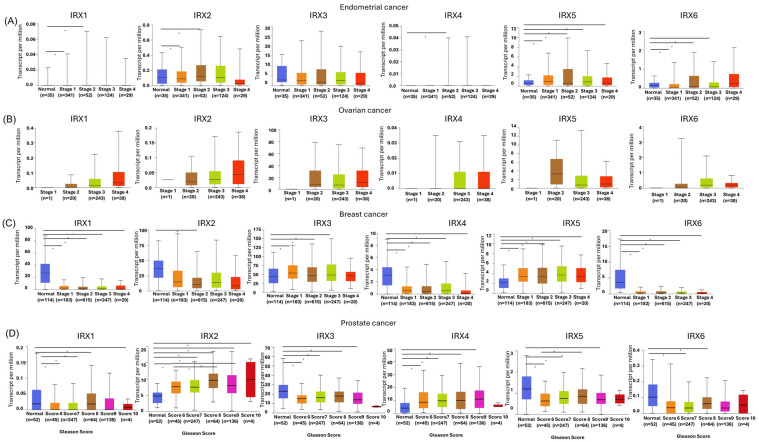
Differential expression of *IRX* family genes in hormone-sensitive cancer progression. (**A**) Expression of *IRX* family members in endometrial cancer stages. (**B**) Expression of *IRX* family members in ovarian serous cystadenocarcinoma stages. (**C**) Expression of *IRX* family members in breast cancer stages. (**D**) Expression of *IRX* family members in prostate cancer Gleason scores (UALCAN database, TPM RNA seq data from TCGA, * *p* < 0.05).

Using TCGA data accessed through UALCAN, we analyzed *IRX* gene expression across tumour stages in primary hormone-sensitive cancers and corresponding adjacent normal tissues. In endometrial and ovarian cancers, *IRX3* and *IRX5* expression remained relatively consistent across tumour stages, with no significant stage-dependent changes observed (Figure 3A–B). In breast cancer, both *IRX3* and *IRX5* were consistently upregulated compared to normal tissues; however, no clear progressive increase across stages was evident (Figure 3C). In prostate cancer, *IRX3* and *IRX5* generally showed lower expression relative to normal tissue across stages, although *IRX5* demonstrated a significant increase between higher Gleason scores despite remaining below normal expression levels (Figure 3D). *IRX2* and *IRX4* displayed prostate-specific associations with tumour grade. *IRX2* expression increased with higher Gleason scores, reaching statistical significance, whereas *IRX4* showed a similar trend without consistent statistical significance. In contrast, *IRX2* expression was significantly reduced in early stages of endometrial and breast cancers compared with normal tissues, with differences becoming less pronounced at later stages. *IRX4* maintained significantly lower expression in breast cancer tissues across all stages relative to normal tissue and was also expressed at very low levels in endometrial cancers. *IRX1* exhibited consistently reduced expression throughout tumour stages in both breast and prostate cancers compared to normal tissues and remained minimal in endometrial samples. *IRX6* expression was significantly decreased across tumour stages in breast cancer relative to normal tissue, while in prostate cancer significant reductions were mainly limited to early stages. Conversely, *IRX6* showed a significant increase in early-stage endometrial cancer only.

Overall, these findings indicate that *IRX*3 and *IRX*5 show stable high expression across tumour stages in estrogen-dependent cancers, while *IRX*2 and *IRX*4 exhibit prostate cancer-specific expression patterns associated with disease grade. These patterns suggest potential associations between *IRX* gene expression and tumour characteristics in a cancer type-specific manner.

### 3.4. Prognostic Significance of IRX Genes Across Cancer Types

**Figure 4:**
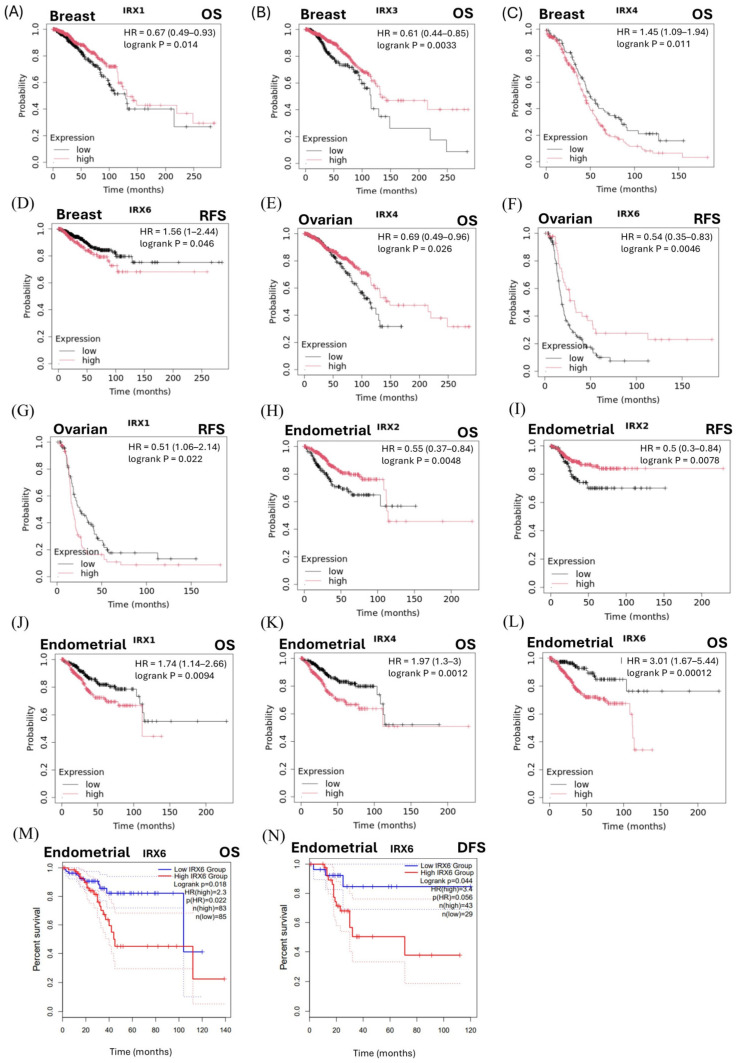
Association of *IRX* gene expression with survivability in hormone-sensitive cancers. Kaplan–Meier survival plots of breast, ovarian, endometrial and prostate cancer with high and low *IRX* expression according to Kaplan–Meier Plotter database (**A**–**L**) and GEPIA database (**M**,**N**). (**A**–**D**) Kaplan–Meier plots for *IRX*1 and *IRX*3 in OS and for *IRX*6 in RFS of breast cancer. (**E**–**G**) Kaplan–Meier plot for *IRX*4, *IRX*6 and *IRX*1 in RFS of ovarian cancer. (**H**–**N**) Kaplan–Meier plots for *IRX*2 and *IRX*6 in OS, RFS and DFS in endometrial cancer.

To evaluate the prognostic significance of *IRX* genes, we performed survival analyses using multiple publicly available databases for hormone-sensitive cancer patients. Analysis using the Kaplan–Meier Plotter indicated that the high expression of *IRX*1 and *IRX*3 was associated with improved OS, whereas elevated expression of *IRX*4 and *IRX*6 was associated with poorer OS and RFS in breast cancer (Figure 4A–D). In ovarian cancer, higher expression of *IRX*4 and *IRX*6 was associated with more favourable survival outcomes, while increased *IRX*1 expression correlated with poorer prognosis (Figure 4E–G). In endometrial cancer, elevated *IRX*2 expression was associated with improved OS and RFS, whereas higher expression of *IRX*1, *IRX*4, and *IRX*6 correlated with poorer prognosis (Figure 4H–L). Consistent with these findings, analysis using the GEPIA database demonstrated that high *IRX*6 expression was associated with reduced OS and DFS in endometrial cancer (Figure 4M,N).

Additional analyses using the PrognoScan database revealed that higher *IRX*2 expression was associated with improved survival in breast and ovarian cancers, while increased *IRX*3 expression was associated with improved survival in ovarian cancer (Supplementary Table S3). In contrast, *IRX*5 expression was associated with poorer prognosis in both breast and ovarian cancers (Supplementary Table S3). PrognoScan analyses further demonstrated context-dependent prognostic associations for *IRX*4 in breast cancer, with high *IRX*4 expression correlating with poor distant metastasis-free survival in one dataset but favourable distant metastasis-free survival in another cohort. Consistently, higher *IRX*4 expression was associated with improved relapse-free survival in breast cancer. In ovarian cancer, however, elevated *IRX*4 expression was significantly associated with poorer overall survival. Notably, no significant associations between *IRX* gene expression and patient survival were observed in prostate cancer across either the GEPIA or PrognoScan datasets. Additional non-significant associations identified across Kaplan–Meier Plotter, GEPIA, and PrognoScan analyses for OS, DFS, and RFS are summarized in Supplementary Figures S2 and S3 and Supplementary Table S3.

### 3.5. Differential Hormonal Regulation of IRX Genes

Given the similar expression patterns of *IRX*3 and *IRX*5 in estrogen-responsive cancers, and *IRX*2 and *IRX*4 in androgen-responsive cancers, we investigated their potential hormonal regulation. We analyzed ChIP-seq datasets for hormone-sensitive cancer patients as well as for their cell lines and performed hormone treatment assays to assess transcriptional control in hormone-sensitive cancer cell lines.

**Figure 5:**
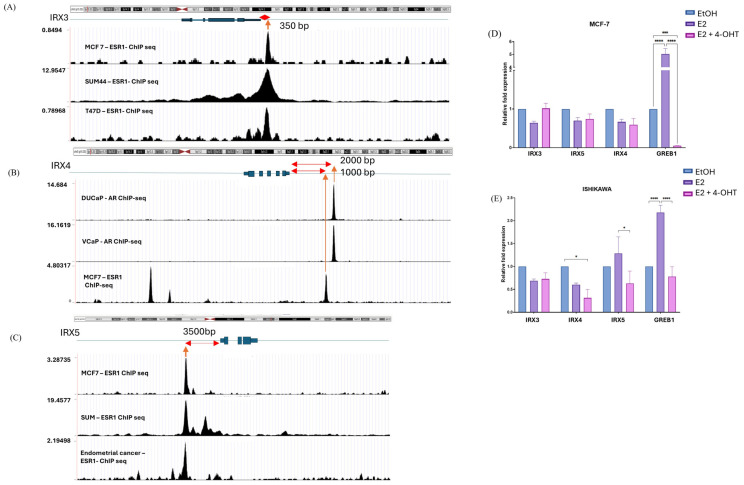
Hormonal regulation of *IRX* genes in hormone-sensitive cancers. (**A**) ESR1 binding peaks in the locus including *IRX*3, (**B**) ESR1 and AR binding peaks in the locus including *IRX*4, (**C**) ESR1 binding peaks in the locus including *IRX*5 (Cistrome Finder: http://cistrome.org/finder, (accessed on 25 May 2024) figure derived from UCSC Genome Browser (accessed on 27 May 2024). Red arrows indicate the distance from the gene promoter to the ChIP-seq peak, while orange arrows mark the ChIP-seq peak location. (**D**) *IRX3*, *4* and *5* expressions with treatment of EtOH, E2 and E2 + 4-OHT in MCF-7 cells. (**E**) *IRX3*, *4* and *5* expressions with treatment of EtOH, E2 and E2 + 4-OHT) in ISHIKAWA cells. Relative fold expression of *IRX*s compared to EtOH/control expression was measured using the ΔΔCT method using RPL32 as the endogenous control. (n = 3 biological and n = 3 technical, two-way ANOVA test with Tukey’s multiple comparisons, mean ± SEM, * *p* < 0.05, ** *p* < 0.01, *** *p* < 0.001, **** *p* < 0.0001).

We examined their potential regulation by major estrogen- and androgen-activated transcription factors including ESR1 and AR through ChIP-seq binding peak analysis using Cistrome data. We identified an ESR1 binding peak within 350 bp of the *IRX*3 promoter in MCF-7, T47D, and SUM44 breast cancer cell lines (Figure 5A). Additionally, ESR1 binding peaks were observed 1000 bp and 2000 bp upstream of the *IRX*4 promoter in MCF-7 cells, suggesting ESR1-dependent regulation in breast cancer, and AR binding peaks were detected at similar distances from the *IRX*4 promoter in DuCaP and VCaP prostate cancer cell lines, suggesting its AR-dependent regulation in prostate cancer (Figure 5B). Furthermore, an ESR1 peak was detected 3000 bp upstream of the *IRX*5 promoter in breast cancer cell lines including MCF-7, SUM44, and endometrial cancer cell lines, suggesting potential estrogen regulation of *IRX*5 in breast and endometrial cancer (Figure 5C). Overall ChIP-seq data suggested that the *IRX*3, *IRX*4 and *IRX*5 genes were prone to being differentially hormone-regulated in estrogen- and androgen-dependent cancers.

To confirm the estrogen dependency of these *IRX*s, next we performed an estrogen treatment assay in hormone-sensitive cancer cell lines. Androgen-mediated regulation of alternative transcripts of *IRX4* was previously shown by our group, supporting the observed androgen regulation of *IRX4* in prostate cancer [29]. Thus, the expression of each *IRX3* and *IRX5* was determined in estrogen-responsive cell lines (MCF-7, ISHIKAWA) with estrogen (E2) and anti-estrogen (4-hydroxytamoxifen) treatment. GREB1 expression was used as a positive control to breast and endometrial cancer to validate both the treatments [43]. GREB1 was overexpressed with E2 treatment in both cell lines (MCF-7 ~4.0-fold and ISHIKAWA ~1.5 fold) and downregulated with anti-estrogen treatment compared to the ethanol (EtOH) control. Despite robust responses of GREB1 with E2 and 4-hydroxytamoxifen in the MCF-7 and ISHIKAWA cell lines, any of the *IRX* genes that have peaks for ESR1 in their promoter region did not show any significant difference in their expression with E2 treatment. However, *IRX4* and *IRX5* expression was significantly reduced with estrogen receptor inhibitors in the MCF-7 and ISHIKAWA cell lines, suggesting a partial dependency on ER signalling; however, E2 alone did not induce significant expression, indicating that promoter occupancy may be necessary but not sufficient for robust transcriptional activation (Figure 5D,E).

### 3.6. Association of IRX Genes with Cancer Stemness and Drug Resistance

**Figure 6:**
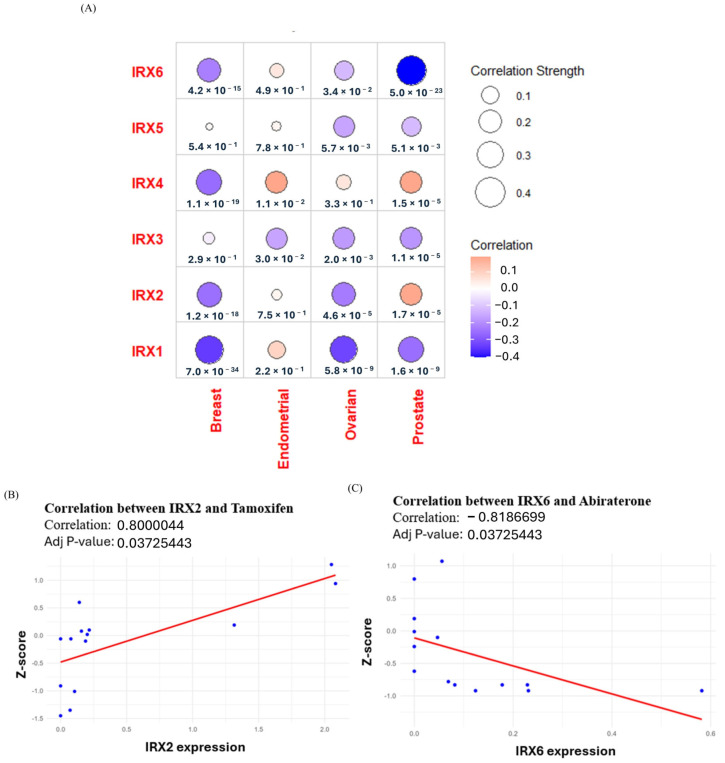
Correlation of *IRX* family with tumour stemness and drug sensitivity in hormone-sensitive cancers. (**A**) Correlation analysis of *IRX* gene expression with tumour RNA stemness scores in breast, endometrial, ovarian and prostate cancers. (**B**) Correlation between *IRX*2 gene expression with 4-OHT (correlation: 0.8000044, adjusted *p*-value: 0.03725443). (**C**) Correlation between *IRX*6 gene expression with abiraterone (correlation: −0.8186699, adjusted *p*-value: 0.03725443). Blue dots represent individual cell lines, and the red line indicates the fitted linear regression line used to assess the correlation.

*IRX* family genes, which are involved in developmental processes and have been linked to stemness-associated signalling pathways, have also been implicated in therapy resistance through promoting cancer stemness in several cancers [8,44,45]. To explore this association, we analyzed correlations between *IRX* gene expression and RNA-based stemness score values in breast, ovarian, endometrial, and prostate cancer patients using TCGA data from the UCSC Xena database [42]. This analysis revealed cancer type-specific patterns. In breast cancer, *IRX1*, *IRX2*, *IRX4*, and *IRX5* expression showed significant negative correlations with stemness scores (Figure 6A). In endometrial cancer, only *IRX4* demonstrated a positive correlation with stemness. In ovarian cancer, all *IRX* genes except *IRX4* were negatively correlated with stemness. In contrast, in prostate cancer, *IRX2* and *IRX4* expression showed positive correlations with stemness scores (Figure 6A).

To further examine potential clinical associations, we assessed correlations between *IRX* gene expression and drug sensitivity using hormone-sensitive cancer cell lines from the NCI-60 panel via the CellMiner database. Overall, few significant associations were identified; however, two correlations remained significant after adjustment for multiple testing (adjusted *p* < 0.05). *IRX2* expression was positively correlated with sensitivity to 4-OHT (Figure 6B), while *IRX6* expression was negatively correlated with sensitivity to abiraterone (Figure 6C). All correlation coefficients between *IRX* gene expression and drug responses in hormone-sensitive cancers are provided in Supplementary Table S4.

Together, these analyses demonstrate context-dependent associations between *IRX* gene expression, stemness-related features, and drug response across hormone-sensitive malignancies.

## 4. Discussion

The *IRX* family of homeobox transcription factors, originally discovered in Drosophila, is conserved across metazoans and plays essential roles in embryonic development, governing cell fate specification and organogenesis in both vertebrates and invertebrates [10,11]. The dysregulation of *IRX* genes has been increasingly implicated in oncogenesis, with reports of their involvement in tumour promotion or suppression in diverse malignancies, including prostate cancer, head and neck carcinoma, and hepatocellular carcinoma [12,13,29,46]. Genome-wide association studies have further linked loci encompassing *IRX* genes to cancer susceptibility mostly for hormone-sensitive cancers, such as the *5p15* region associated with prostate cancer and the *16q12* region housing Cluster B *IRX* genes linked to breast cancer risk [22,23,24,26,27,47]. Despite these observations, the expression patterns and clinical relevance of *IRX* genes in hormone-sensitive cancers have remained incompletely characterized.

In this study, we performed a comprehensive, pan-cancer analysis of all six *IRX* genes (*IRX1*–*IRX6*) across prostate, breast, ovarian, and endometrial cancers using large-scale publicly available datasets, complemented by limited in vitro validation. Our analyses revealed distinct and context-dependent expression patterns of *IRX* genes across hormone-sensitive cancers, highlighting heterogeneous associations with tumour characteristics, patient outcomes, cancer stemness-related features, and therapeutic response.

A key finding of our study is the consistent overexpression of *IRX*3 and *IRX*5 in estrogen-sensitive cancers, including breast, ovarian, and endometrial cancers. Previous research has implicated these genes as oncogenic drivers, promoting cellular proliferation, migration, and invasion in diverse tumour types such as hepatocellular carcinoma, breast cancer, tongue squamous cell carcinoma, and non-small cell lung cancer [14,16,46,48]. Moreover, co-expression and functional cooperation between *IRX3* and *IRX5* have been described in Wilms tumours and colorectal cancer, suggesting coordinated regulation within the *IRX* gene clusters [49,50]. The conserved three-dimensional chromatin organization of *IRX* clusters comprising *IRX1/IRX2* (Cluster A) and *IRX3/IRX5* (Cluster B) supports the possibility of shared regulatory elements contributing to coordinated expression [51]. Interestingly, despite these oncogenic associations reported in other tumour contexts, our survival analyses indicated that higher *IRX3* expression was associated with more favourable outcomes in breast and ovarian cancers, whereas *IRX5* was more frequently associated with poorer prognosis. These findings underscore the context and tumour type-specific nature of *IRX* gene associations and suggest that elevated *IRX3* expression in estrogen-dependent cancers may reflect a more differentiated or hormone-responsive tumour state rather than aggressive behaviour. Such heterogeneity highlights the importance of considering tumour context, disease stage, and molecular background when interpreting *IRX* gene expression patterns.

*IRX2* and *IRX4* displayed particularly divergent associations across hormone-sensitive cancers, despite residing within the same genomic cluster on chromosome 5. *IRX2* was significantly upregulated in prostate cancer and showed associations with higher Gleason scores, suggesting a link with more advanced disease features. Similar associations between *IRX2* expression and tumour progression have been reported in soft tissue sarcomas [52]. In contrast, in breast cancer, *IRX*2 was downregulated relative to normal tissues and associated with favourable prognosis and reduced stemness scores. Furthermore, high *IRX2* is associated with favourable prognosis in ovarian and endometrial cancers with a negative correlation with cancer stemness score in ovarian cancer too, suggesting features consistent with a tumour-suppressive association. These observations are consistent with reports of the epigenetic silencing of *IRX2* in other tumour types, including promoter hypermethylation in non-small cell lung cancer and the derepression of oncogenic pathways following *IRX2* downregulation in endometrial cancer [53,54]. *IRX4* exhibited a contrasting pattern, with increased expression in prostate, ovarian, and endometrial cancers but reduced expression in breast cancer. Overall, *IRX4* expression was more frequently associated with poorer clinical outcomes; however, notable inconsistencies were observed across datasets, with some cohorts demonstrating favourable associations with relapse-free or distant metastasis-free survival. These discrepancies likely reflect cohort heterogeneity, differences in tumour subtype, treatment history, and expression cutoff thresholds. Previous studies linking genetic variants at the 5p15.33 locus to *IRX4* expression and prostate cancer susceptibility further support a regulatory role for this gene in prostate malignancy [15]. Furthermore, the aberrant methylation of the *IRX4* promoter has been shown to reduce *IRX*4 expression, supporting its potential tumour-suppressive role in cancer. Collectively, these findings suggest that *IRX2* and *IRX4*, while physically clustered, are subject to divergent regulatory mechanisms and exhibit distinct, context-dependent associations in hormone-sensitive cancers.

*IRX*1 and *IRX*6 generally showed lower expression across the cancer types examined, yet both were notably upregulated in ovarian cancer and significantly downregulated in breast and prostate cancers. *IRX*1 has been characterized as a tumour suppressor in gastric cancer, where reduced expression is linked to promoter hypermethylation and increased tumorigenicity [24]. *IRX6* remains comparatively understudied in cancer; while higher *IRX6* expression has been associated with favourable outcomes in lung adenocarcinoma [55], our analyses revealed associations between elevated *IRX6* expression and poorer prognosis in endometrial cancer, highlighting a potentially context-specific role for *IRX6* in tumour biology.

Given the shared expression patterns of several *IRX* genes in hormone-sensitive cancers, we investigated their potential regulation by sex steroid hormones. ChIP-seq analysis identified ESR1 binding peaks near *IRX*3, *IRX*4, and *IRX*5 promoters in breast cancer cell lines, yet our estrogen treatment assays in ER-positive MCF-7 and Ishikawa cells revealed no significant changes in *IRX* gene expression following estrogen stimulation or inhibition. These results underscore that ESR1 binding alone is insufficient to drive transcriptional activation, likely due to factors such as chromatin accessibility, the absence of necessary co-factors, or poised enhancer states that remain inactive under certain conditions [56,57]. However, it is important to note that our analyses were limited to specific datasets and cell lines, and the regulatory effects of estrogen on *IRX* genes may become evident in other cellular contexts or with larger datasets. These observations highlight the complexity of estrogen receptor signalling and demonstrate that transcription factor binding does not necessarily equate to transcriptional activation. In contrast, AR ChIP-seq data in prostate cancer cell lines revealed AR binding peaks near the *IRX*4 promoter, indicating direct androgen regulation, which aligns with the elevated *IRX*4 expression we observed in prostate cancer and prior reports of androgen-regulated *IRX*4 transcripts in VCaP and DuCaP cells [44]. Interestingly, despite *IRX*2’s upregulation in prostate cancer, no AR or ESR1 binding was detected near its regulatory regions, suggesting that alternative transcriptional control mechanisms warrant further exploration.

Building on the observed tumour-specific expression and prognostic associations of *IRX* genes, we next examined their associations with stemness-related features and therapeutic response Our analyses identified context-dependent associations between *IRX* gene expression, cancer stemness-related features, and drug response. *IRX2* expression was negatively correlated with stemness scores in estrogen-dependent cancers and positively correlated with sensitivity to 4-OHT in hormone-sensitive cell lines, consistent with previous reports linking *IRX2* to less aggressive, hormone receptor-positive breast cancer subtypes [30]. Conversely, in prostate cancer, *IRX*2, and *IRX4* expression showed a positive correlation with stemness scores, implicating it in maintaining cancer stem cell populations in androgen-driven contexts. Notably, we observed a novel negative correlation between *IRX6* expression and sensitivity to abiraterone, suggesting that elevated *IRX6* expression may be associated with a reduced response to androgen biosynthesis inhibition in certain contexts. While these findings are exploratory, they highlight potential links between *IRX* gene expression and treatment response that merit further investigation.

Several limitations should be considered when interpreting this work. First, much of this study is based on integrative analyses across multiple public resources, each with distinct preprocessing pipelines, cohort compositions, and statistical approaches. Second, our workflow involves numerous comparisons across genes, cancers, endpoints, and databases; therefore, without harmonized false discovery control across analytic blocks, some observed associations may represent false positives. Third, tumour–non-tumour comparisons derived from integrated TCGA/GTEx resources can be affected by residual batch effects and differences in tissue procurement; thus, expression differences should be interpreted primarily in terms of directionality and consistency, rather than as definitive effect size estimates. Fourth, survival analyses were performed using portals that employ different grouping strategies (including quartile-based grouping and data-driven “optimal” cutoffs), which can contribute to variability across cohorts; future work should prioritize a consistent survival modelling strategy with adjustment for clinical covariates and subtype. Finally, experimental validation was intentionally limited in scope and remains insufficient to establish causality or define mechanisms, reinforcing that the present study provides a framework for prioritization rather than a definitive mechanistic model.

## 5. Conclusions

In summary, this study provides a comprehensive analysis demonstrating that *IRX* genes exhibit distinct, context-dependent expression patterns across hormone-sensitive cancers. Our findings indicate that *IRX* family members display heterogeneous associations with tumour characteristics, patient outcomes, cancer stemness-related features, and drug response, which vary according to tumour type and hormonal context. Notably, *IRX*3 and *IRX*5 showed consistent overexpression in estrogen-dependent cancers and were associated with clinical outcomes that differed across tumour types, underscoring the complexity of their context-specific roles. *IRX*2 and *IRX*4 exhibited divergent expression and prognostic associations, particularly in prostate cancer, where their expression correlated with disease grade and stemness-related signatures. In contrast, in estrogen-driven cancers, *IRX*2 expression was generally associated with more favourable clinical features. *IRX*6, although less well characterized in cancer, demonstrated associations with poor prognosis and reduced sensitivity to abiraterone in specific contexts, highlighting a potential link with treatment response.

Importantly, these findings are primarily based on correlative analyses of publicly available datasets and limited in vitro validation and should therefore be interpreted with caution. While the observed associations suggest that *IRX* genes may have relevance as biomarkers of tumour behaviour and treatment response in hormone-sensitive cancers, further functional and mechanistic studies are required to establish causality and to determine their potential clinical utility. Collectively, this work provides a foundation for future investigations into the context-dependent roles of *IRX* family members in hormone-driven malignancies.

## Data Availability

All the data presented in this study are available in the manuscript. These data were derived from the following resources available in the public domain: Pan-Cancer Cell Line Transcriptome Atlas: https://pcatools.shinyapps.io/PCTA_app/ (accessed on 12 November 2024), TNMplot: https://tnmplot.com/analysis/ (accessed on 25 October 2024), UALCAN: http://ualcan.path.uab.edu (accessed on 24 November 2024), GEPIA: http://gepia2.cancer-pku.cn/#index (accessed on 5 March 2025), Kaplan–Meier Plotter: https://kmplot.com/analysis/ (accessed on 3 March 2025), Cistrome: (http://cistrome.org/db/#/ (accessed on 25 May 2024), Cell Miner: https://discover.nci.nih.gov/cellminer/ (accessed on 30 September 2024), and UCSC XENA: https://xena.ucsc.edu/ (accessed on 15 August 2024). The raw data supporting the conclusions of this article will be made available by the authors on request.

## Supplementary Materials

The following supporting information can be downloaded at: https://www.mdpi.com/article/10.3390/cancers18050726/s1, Figure S1: Differential expression of *IRX*s in hormone sensitive cancer tissues compared to their normal counterparts using TCGA and GEO data; Figure S2: Kaplan-Meier plots for overall survival (OS) and disease free survival (DFS) *IRX* gene expression in hormone sensitive cancers using GEPIA; Figure S3: Kaplan-Meier plots for OS and relapse free survival (RFS) for *IRX* gene expression in hormone sensitive cancers, generated using Kaplan Meier plotter database; Table S1: Primer sequences for qRT-PCR; Table S2: The characteristics of prostate, breast, ovarian and endometrial cancer cell lines used in the study; Table S3: Survival analysis of *IRX*s in hormone sensitive cancers using PrognoScan web tool; Table S4: Correlation coefficient values of *IRX*s with drugs in hormone sensitive cancers.

## Abbreviations

The following abbreviations are used in this manuscript:

IRX	Iroquois class homeobox
CSCs	Cancer stem cells
GWAS	Genome-wide association study
PCTA	Pan-cancer cell line transcriptome atlas
GTEx	Genotype-tissue expression
TCGA	The cancer genomic atlas
OS	Overall survival
RFS	Relapse-free survival
DFS	Disease-free survival
ESR1	Estrogen alpha receptor
AR	Androgen receptor
