# Supplementary material for: Distinct Expression Patterns and Clinical Associations of the IRX Gene Family Across Hormone-Sensitive Cancers
Source: Cancers (Basel). 2026 Feb 24;18(5):726. doi: 10.3390/cancers18050726 (PMC12984367; doi:10.3390/cancers18050726)
Supplement: Supplementary file 1 [file cancers-18-00726-s001.zip › cancers-4138913-supplementary.pdf]

Figure S1: Differential expression of IRXs in hormone sensitive cancer tissues compared to their normal counterparts using TCGA and GEO data. (Unpaired t-test, mean ± SD, * $p < 0.05$, ** $p < 0.01$, *** $p < 0.001$, **** $p < 0.0001$).

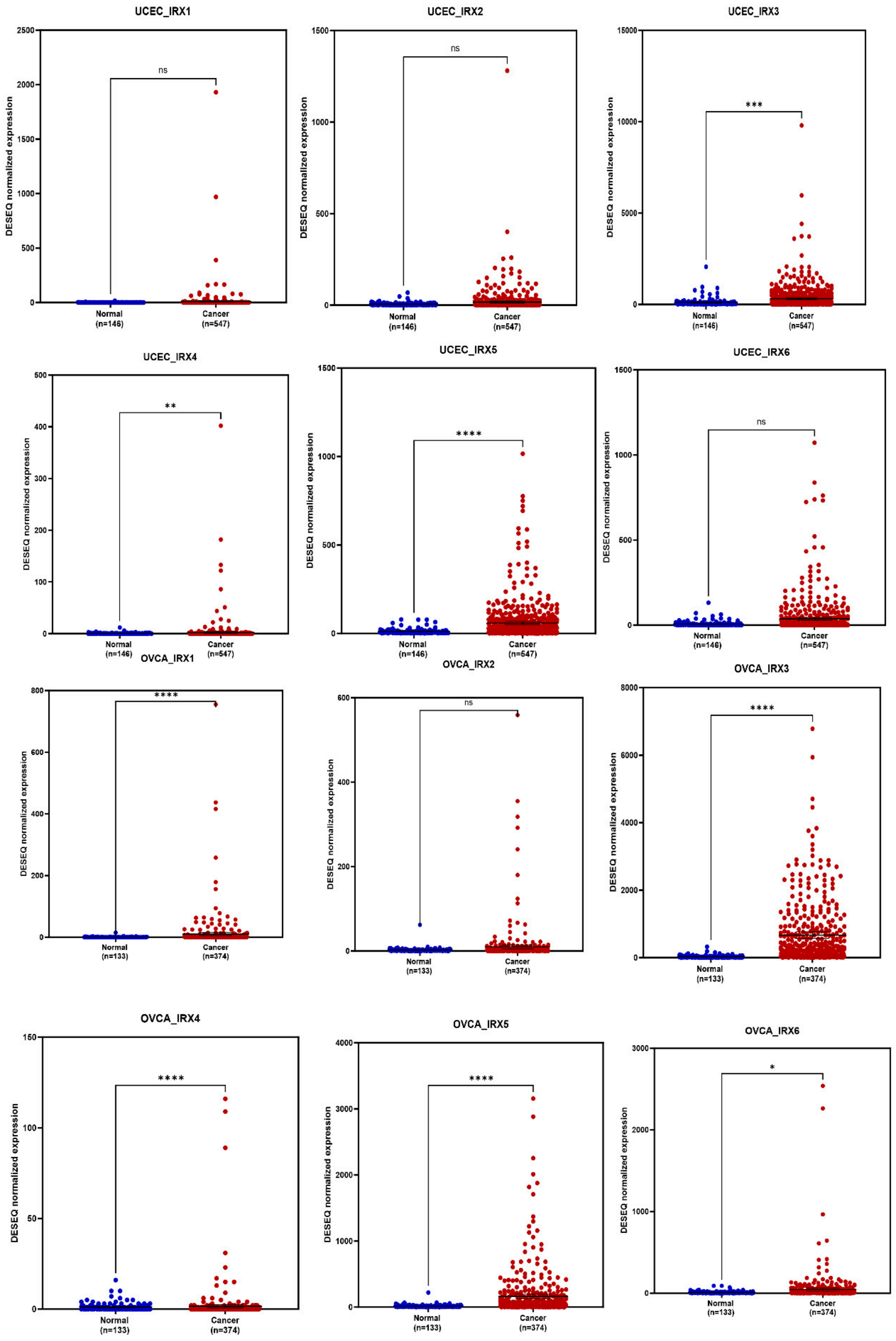

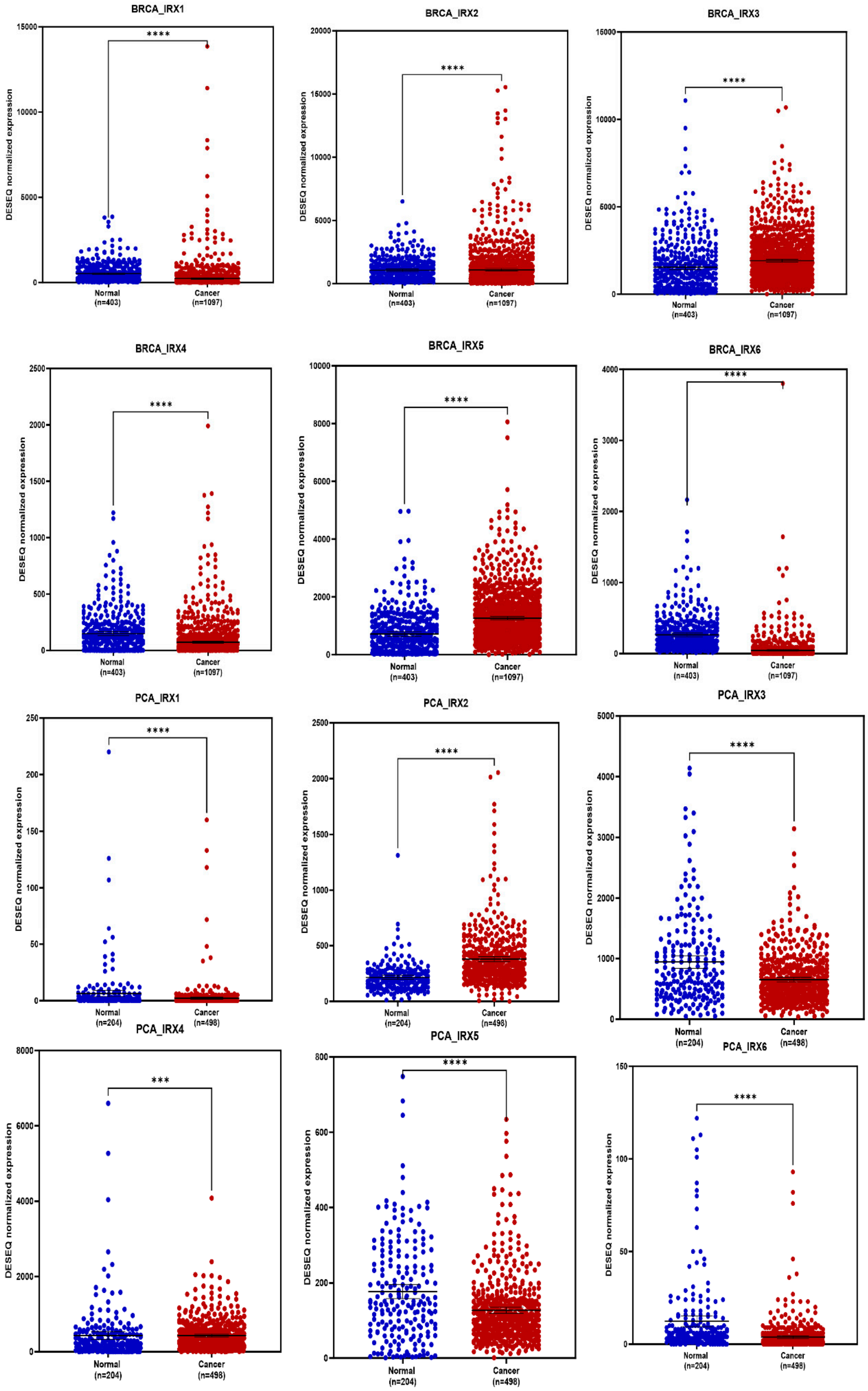
BRCA_IRX1
BRCA_IRX2
BRCA_IRX3
BRCA_IRX4
BRCA_IRX5
BRCA_IRX6
PCA_IRX1
PCA_IRX2
PCA_IRX3
PCA_IRX4
PCA_IRX5
PCA_IRX6
DESEQ normalized expression
****
***
Normal
(n=403)
Cancer
(n=1097)
Normal
(n=204)
Cancer
(n=498)

Figure S2: Kaplan-Meier plots for overall survival (OS) and disease-free survival (DFS) IRX gene expression in hormone sensitive cancers using GEPIA.

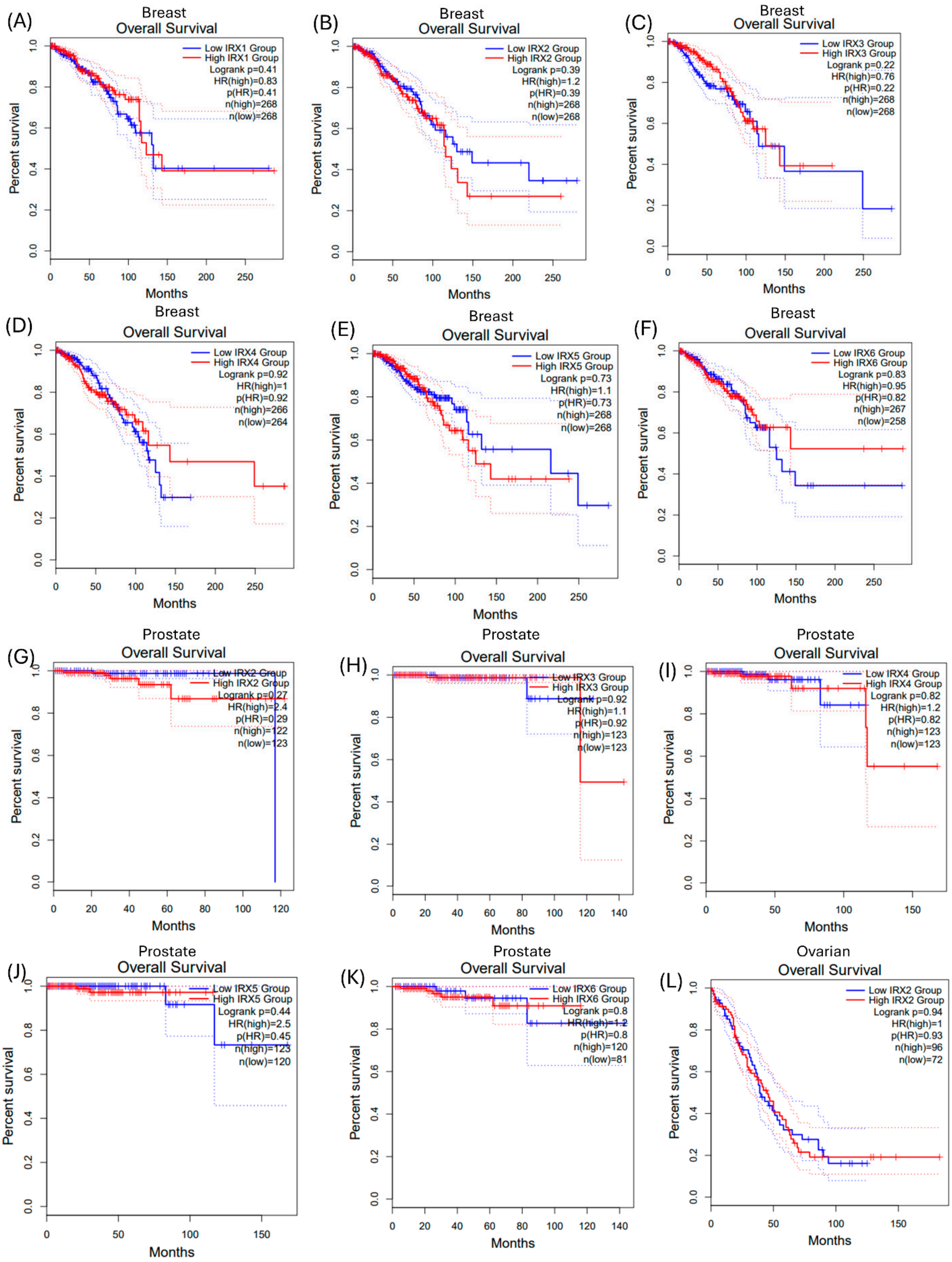

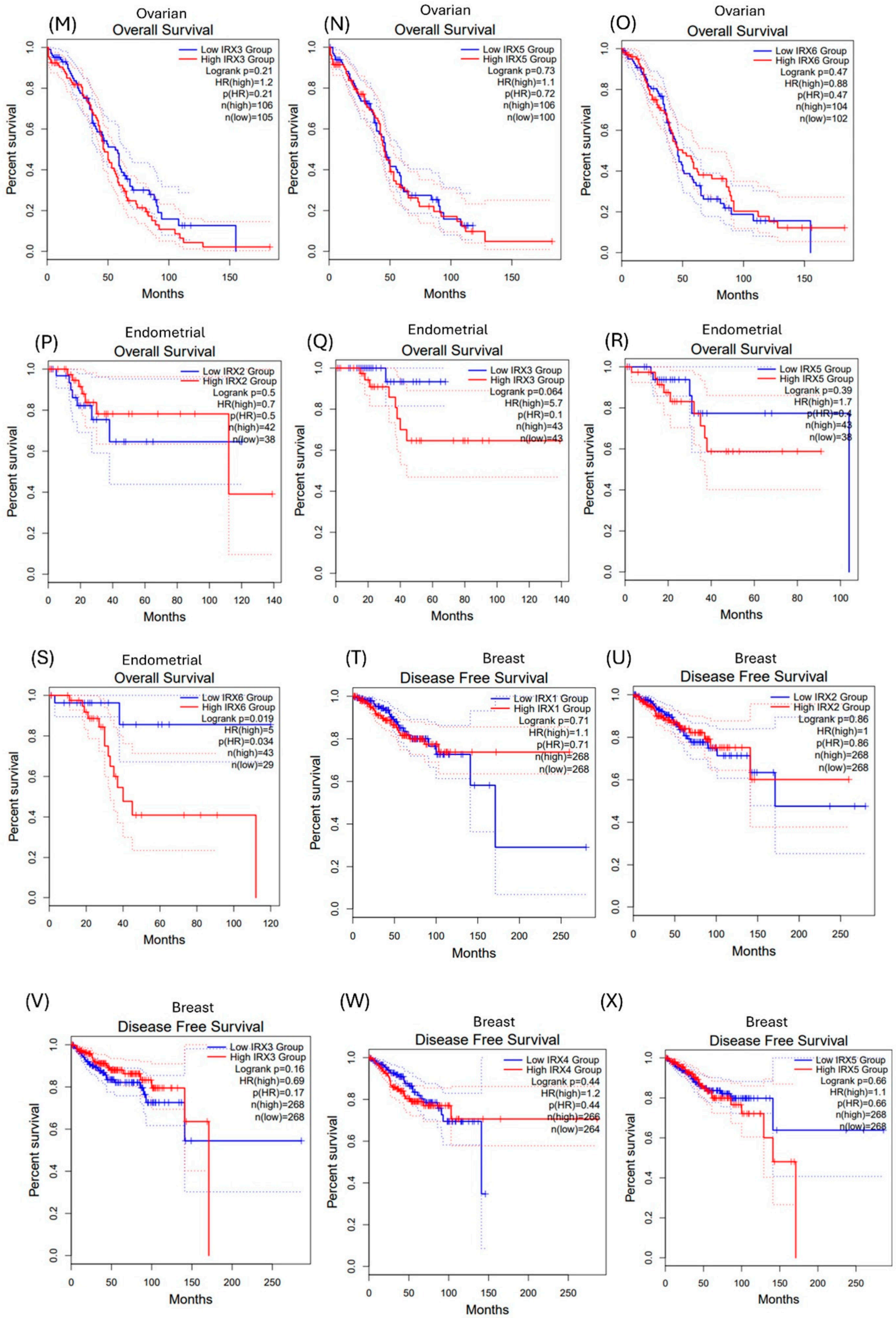
(M)
Ovarian
Overall Survival
Low IRX3 Group
High IRX3 Group
Logrank p=0.21
HR(high)=1.2
p(HR)=0.21
n(high)=106
n(low)=105
Percent survival
Months
(N)
Ovarian
Overall Survival
Low IRX5 Group
High IRX5 Group
Logrank p=0.73
HR(high)=1.1
p(HR)=0.72
n(high)=106
n(low)=100
Percent survival
Months
(O)
Ovarian
Overall Survival
Low IRX6 Group
High IRX6 Group
Logrank p=0.47
HR(high)=0.88
p(HR)=0.47
n(high)=104
n(low)=102
Percent survival
Months
(P)
Endometrial
Overall Survival
Low IRX2 Group
High IRX2 Group
Logrank p=0.5
HR(high)=0.7
p(HR)=0.5
n(high)=42
n(low)=38
Percent survival
Months
(Q)
Endometrial
Overall Survival
Low IRX3 Group
High IRX3 Group
Logrank p=0.064
HR(high)=5.7
p(HR)=0.1
n(high)=43
n(low)=43
Percent survival
Months
(R)
Endometrial
Overall Survival
Low IRX5 Group
High IRX5 Group
Logrank p=0.39
HR(high)=1.7
p(HR)=0.4
n(high)=43
n(low)=38
Percent survival
Months
(S)
Endometrial
Overall Survival
Low IRX6 Group
High IRX6 Group
Logrank p=0.019
HR(high)=5
p(HR)=0.034
n(high)=43
n(low)=29
Percent survival
Months
(T)
Breast
Disease Free Survival
Low IRX1 Group
High IRX1 Group
Logrank p=0.71
HR(high)=1.1
p(HR)=0.71
n(high)=268
n(low)=268
Percent survival
Months
(U)
Breast
Disease Free Survival
Low IRX2 Group
High IRX2 Group
Logrank p=0.86
HR(high)=1
p(HR)=0.86
n(high)=268
n(low)=268
Percent survival
Months
(V)
Breast
Disease Free Survival
Low IRX3 Group
High IRX3 Group
Logrank p=0.16
HR(high)=0.69
p(HR)=0.17
n(high)=268
n(low)=268
Percent survival
Months
(W)
Breast
Disease Free Survival
Low IRX4 Group
High IRX4 Group
Logrank p=0.44
HR(high)=1.2
p(HR)=0.44
n(high)=266
n(low)=264
Percent survival
Months
(X)
Breast
Disease Free Survival
Low IRX5 Group
High IRX5 Group
Logrank p=0.66
HR(high)=1.1
p(HR)=0.66
n(high)=268
n(low)=268
Percent survival
Months

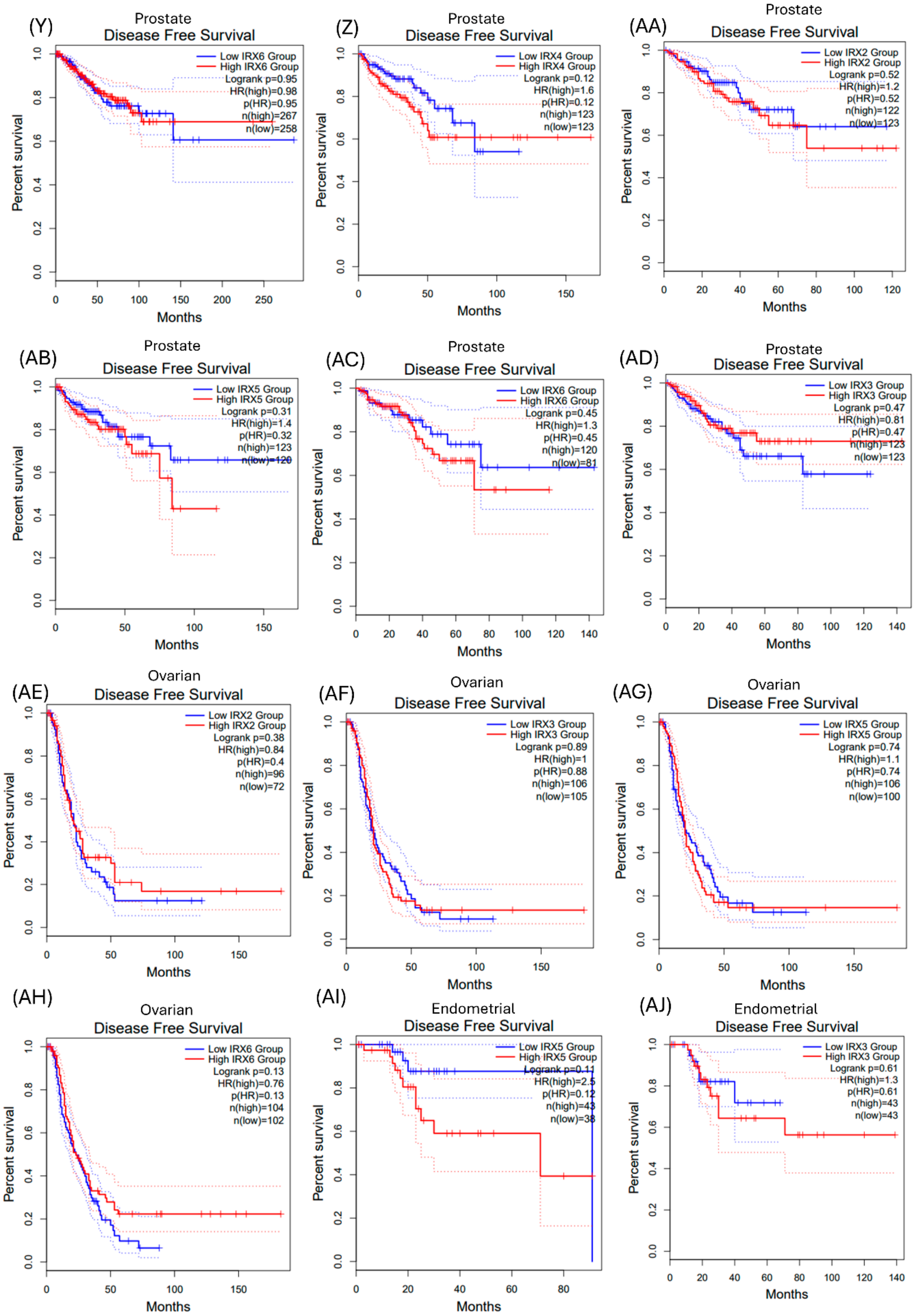
(Y)
Prostate
Disease Free Survival
Low IRX6 Group
High IRX6 Group
Logrank p=0.95
HR(high)=0.98
p(HR)=0.95
n(high)=267
n(low)=258
Percent survival
Months
(Z)
Prostate
Disease Free Survival
Low IRX4 Group
High IRX4 Group
Logrank p=0.12
HR(high)=1.6
p(HR)=0.12
n(high)=123
n(low)=123
Percent survival
Months
(AA)
Prostate
Disease Free Survival
Low IRX2 Group
High IRX2 Group
Logrank p=0.52
HR(high)=1.2
p(HR)=0.52
n(high)=122
n(low)=123
Percent survival
Months
(AB)
Prostate
Disease Free Survival
Low IRX5 Group
High IRX5 Group
Logrank p=0.31
HR(high)=1.4
p(HR)=0.32
n(high)=123
n(low)=120
Percent survival
Months
(AC)
Prostate
Disease Free Survival
Low IRX6 Group
High IRX6 Group
Logrank p=0.45
HR(high)=1.3
p(HR)=0.45
n(high)=120
n(low)=81
Percent survival
Months
(AD)
Prostate
Disease Free Survival
Low IRX3 Group
High IRX3 Group
Logrank p=0.47
HR(high)=0.81
p(HR)=0.47
n(high)=123
n(low)=123
Percent survival
Months
(AE)
Ovarian
Disease Free Survival
Low IRX2 Group
High IRX2 Group
Logrank p=0.38
HR(high)=0.84
p(HR)=0.4
n(high)=96
n(low)=72
Percent survival
Months
(AF)
Ovarian
Disease Free Survival
Low IRX3 Group
High IRX3 Group
Logrank p=0.89
HR(high)=1
p(HR)=0.88
n(high)=106
n(low)=105
Percent survival
Months
(AG)
Ovarian
Disease Free Survival
Low IRX5 Group
High IRX5 Group
Logrank p=0.74
HR(high)=1.1
p(HR)=0.74
n(high)=106
n(low)=100
Percent survival
Months
(AH)
Ovarian
Disease Free Survival
Low IRX6 Group
High IRX6 Group
Logrank p=0.13
HR(high)=0.76
p(HR)=0.13
n(high)=104
n(low)=102
Percent survival
Months
(AI)
Endometrial
Disease Free Survival
Low IRX5 Group
High IRX5 Group
Logrank p=0.11
HR(high)=2.5
p(HR)=0.12
n(high)=43
n(low)=38
Percent survival
Months
(AJ)
Endometrial
Disease Free Survival
Low IRX3 Group
High IRX3 Group
Logrank p=0.61
HR(high)=1.3
p(HR)=0.61
n(high)=43
n(low)=43
Percent survival
Months

(AK)

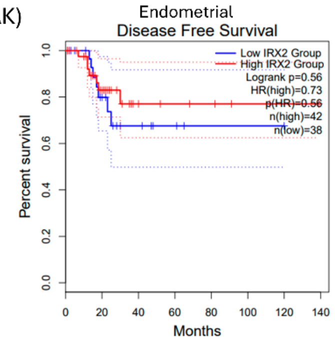
Endometrial
Disease Free Survival
Low IRX2 Group
High IRX2 Group
Logrank p=0.56
HR(high)=0.73
p(HR)=0.56
n(high)=42
n(low)=38
Percent survival
0.0
0.2
0.4
0.6
0.8
1.0
0
20
40
60
80
100
120
140
Months

Figure S3: Kaplan-Meier plots for overall survival (OS) and relapse free survival (RFS) for IRX gene expression in hormone sensitive cancers, generated using Kaplan Meier plotter database.

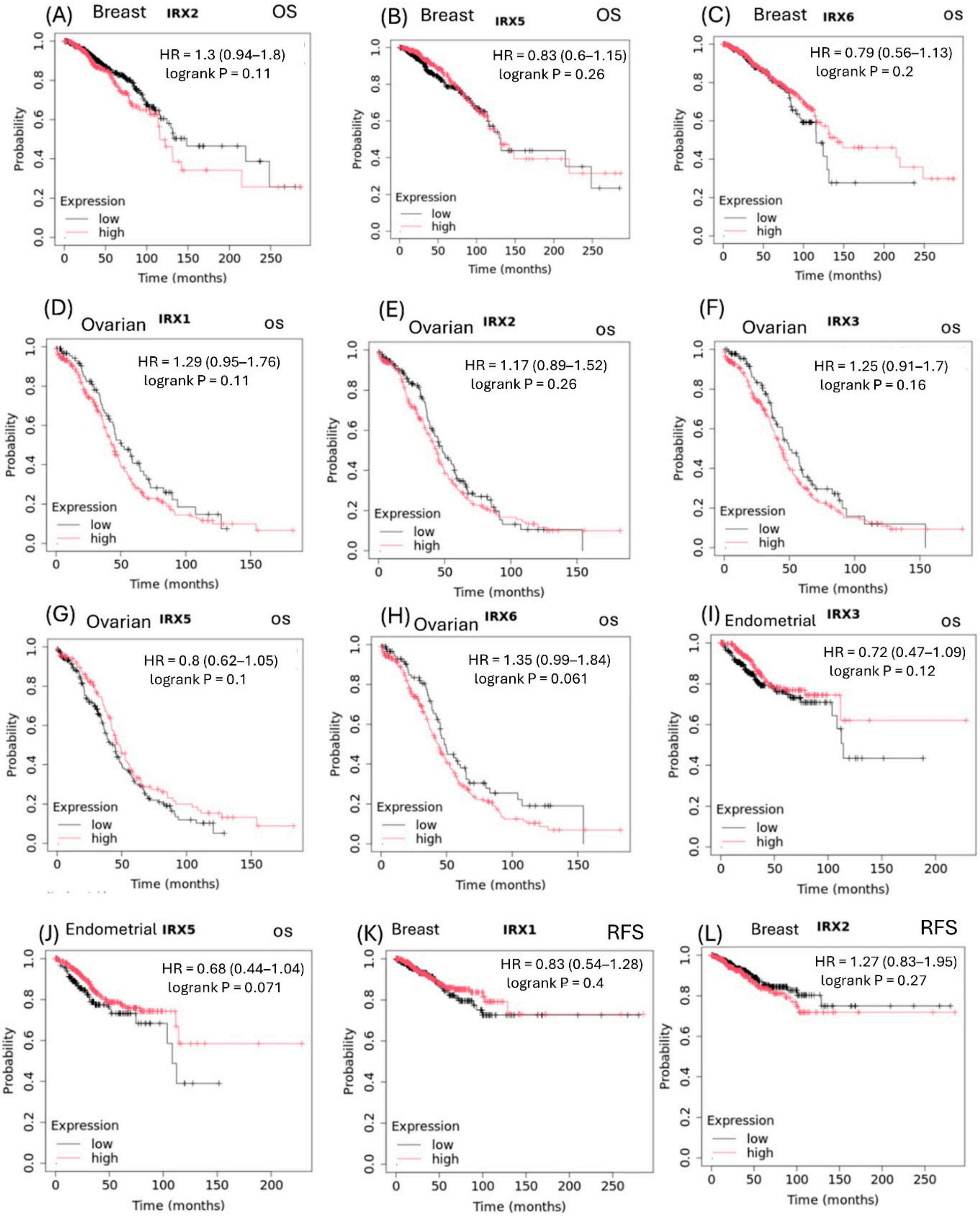

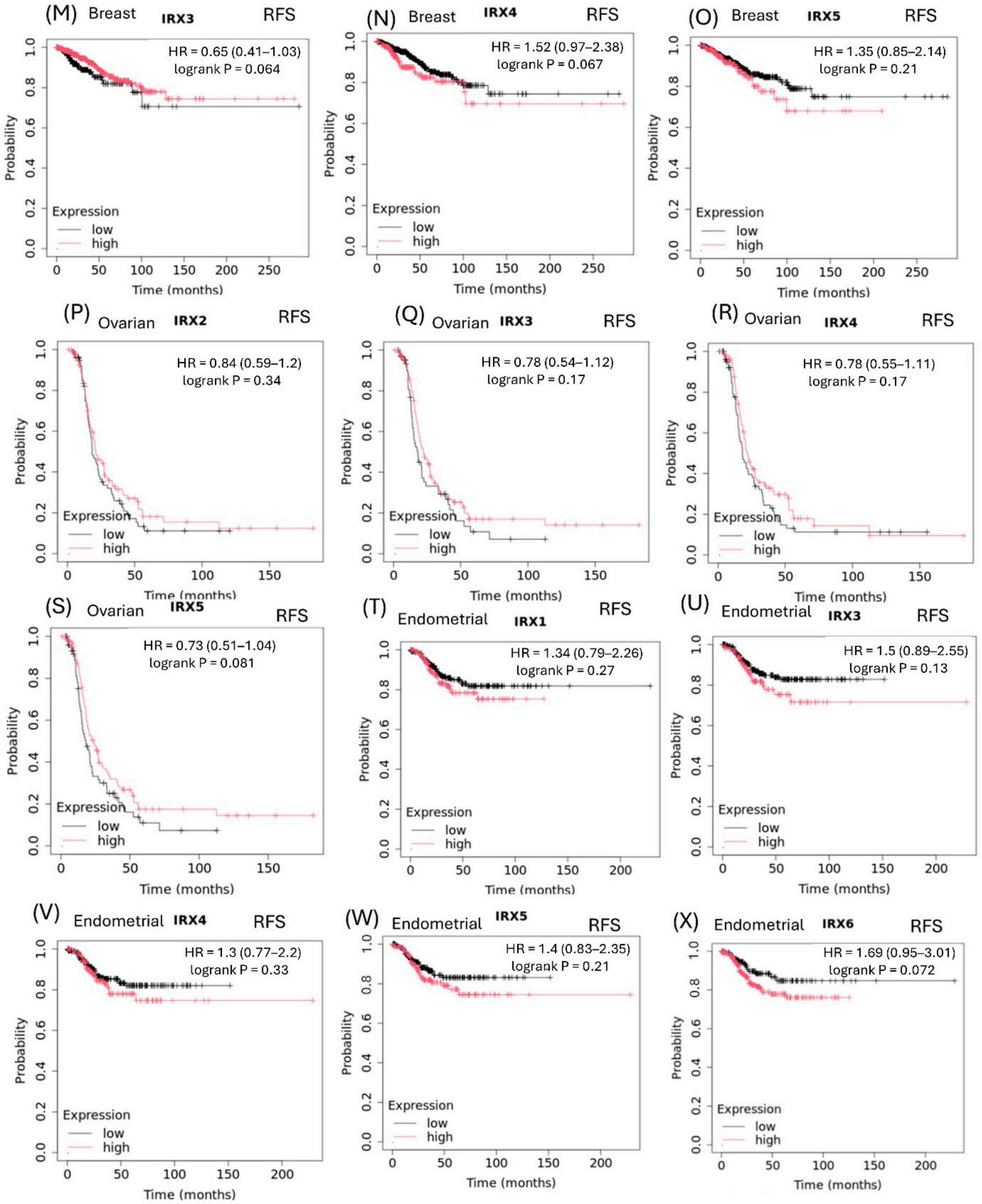

(M) Breast IRX3 RFS
HR = 0.65 (0.41–1.03)
logrank P = 0.064
(N) Breast IRX4 RFS
HR = 1.52 (0.97–2.38)
logrank P = 0.067
(O) Breast IRX5 RFS
HR = 1.35 (0.85–2.14)
logrank P = 0.21
(P) Ovarian IRX2 RFS
HR = 0.84 (0.59–1.2)
logrank P = 0.34
(Q) Ovarian IRX3 RFS
HR = 0.78 (0.54–1.12)
logrank P = 0.17
(R) Ovarian IRX4 RFS
HR = 0.78 (0.55–1.11)
logrank P = 0.17
(S) Ovarian IRX5 RFS
HR = 0.73 (0.51–1.04)
logrank P = 0.081
(T) Endometrial IRX1 RFS
HR = 1.34 (0.79–2.26)
logrank P = 0.27
(U) Endometrial IRX3 RFS
HR = 1.5 (0.89–2.55)
logrank P = 0.13
(V) Endometrial IRX4 RFS
HR = 1.3 (0.77–2.2)
logrank P = 0.33
(W) Endometrial IRX5 RFS
HR = 1.4 (0.83–2.35)
logrank P = 0.21
(X) Endometrial IRX6 RFS
HR = 1.69 (0.95–3.01)
logrank P = 0.072
Probability
Time (months)
Expression
low
high

Supplementary Table S1: Primer sequences for qRT-PCR

Gene name	Forward primer sequence (5′ -> 3′)	Reverse primer sequence (5′ -> 3′)
IRX1	GGGGCACTCAATGGAGACAA	GCGACTTTAACTGCTGCTGTGCC
IRX2	CCACCGGCTTCGGGAG	CCGTACGGGTGGTAGCTGAT
IRX3	GATCGCTGTAGTGCCTTGGA	CAGATGGTTCTGGGGCCG
IRX4	AAGGATGGTTCGGGATCTGC	GCATGATCTTCTCGCCCTTG
IRX5	GCTACAACTCGCACCTCCAG	GCGTAAGGATGGTACCCAA
IRX6	GGAACAGCTGGGCTGAGAC	CATGTGGTGCTGGAACTTGC

Supplementary Table S2: The characteristics of prostate, breast, ovarian and endometrial cancer cell lines used in the study.

Cell	Cell type	Characteristics
LNCaP	Prostate cancer (Androgen Responsive)	Derived from left supraclavicular lymph node from a 50-year-old Caucasian male with metastatic prostate carcinoma
C42B	Prostate cancer (Androgen nonresponsive)	Derived from LNCaP cells, Castration-resistant prostate cancer
MCF7	Breast cancer (Hormone responsive)	Derived from the pleural effusion of a 69-year-old Caucasian woman with breast adenocarcinoma
MDA-MB-231	Breast cancer (Hormone nonresponsive)	Derived from pleural effusion (fluid buildup) in a 51-year-old Caucasian female with metastatic mammary adenocarcinoma
SKOV3	Ovarian Cancer	Derived from the ascitic fluid of a 64-year-old Caucasian female with ovarian serous cystadenocarcinoma
CAOV3	Ovarian Cancer (Hormone responsive)	Derived from the primary tumour of a 54-year-old Caucasian female with adenocarcinoma of the ovary.
ISHIKAWA	Endometrial Cancer (Hormone responsive)	Derived from a 39-year-old Japanese patient with a well-differentiated endometrial adenocarcinoma
HEC-1-A	Endometrial Cancer (Hormone nonresponsive)	Derived from the uterus of a 71-year-old, female patient with stage IA endometrial cancer.

Supplementary Table S3: Survival analysis of IRXs in hormone sensitive cancers using PrognoScan web tool

Query	Dataset	Cancer type	Sub type	Endpoint	Cohort	Contributor	Array type	Probe id	N	Cut Point	Minimum p-value	Corrected p-value	Ln (HR high / HR low)	Cox p-value	Ln(HR)	Hr [95% cilow – ciupp]
IRX1	GSE19615	Breast cancer		Distant Metastasis Free Survival	DF/HCC	Li	HG-U133_Plus_2	230472_at	115	0.43	0.073711	-	-0.96	0.904774	-0.04	0.96 [0.52 - 1.78]
IRX1	GSE12276	Breast cancer		Relapse Free Survival	EMC	Bos	HG-U133_Plus_2	230472_at	204	0.86	0.059158	-	0.39	0.922115	-0.01	0.99 [0.88 - 1.12]
IRX1	GSE6532-GPL570	Breast cancer		Distant Metastasis Free Survival	GUYT	Loi	HG-U133_Plus_2	230472_at	87	0.22	0.020417	0.271871	-0.9	0.170052	-0.26	0.77 [0.53 - 1.12]
IRX1	GSE6532-GPL570	Breast cancer		Relapse Free Survival	GUYT	Loi	HG-U133_Plus_2	230472_at	87	0.22	0.020417	0.271871	-0.9	0.170052	-0.26	0.77 [0.53 - 1.12]
IRX1	GSE9195	Breast cancer		Relapse Free Survival	GUYT2	Loi	HG-U133_Plus_2	230472_at	77	0.65	0.02208	0.287222	-2.02	0.091418	-0.39	0.67 [0.43 - 1.07]
IRX1	GSE9195	Breast cancer		Distant Metastasis Free Survival	GUYT2	Loi	HG-U133_Plus_2	230472_at	77	0.77	0.06517	-	-15.48	0.1335	-0.41	0.67 [0.39 - 1.13]

IRX1	GSE1456-GPL97	Breast cancer		Overall Survival	Stockholm (1994-1996)	Pawitan	HG-U133B	230472_at	159	0.67	0.008431	0.141885	-1.11	0.135967	-0.25	0.78 [0.56 - 1.08]
IRX1	GSE1456-GPL97	Breast cancer		Disease Specific Survival	Stockholm (1994-1996)	Pawitan	HG-U133B	230472_at	159	0.65	0.003104	0.06474	-1.62	0.128079	-0.29	0.74 [0.51 - 1.09]
IRX1	GSE1456-GPL97	Breast cancer		Relapse Free Survival	Stockholm (1994-1996)	Pawitan	HG-U133B	230472_at	159	0.65	0.00959	0.156443	-1.03	0.298553	-0.18	0.84 [0.60 - 1.17]
IRX1	GSE3494-GPL97	Breast cancer		Disease Specific Survival	Uppsala (1987-1989)	Miller	HG-U133B	230472_at	236	0.13	0.000229	0.007226	-1.09	0.100457	-0.27	0.77 [0.56 - 1.05]
IRX1	GSE4922-GPL97	Breast cancer		Disease Free Survival	Uppsala (1987-1989)	Ivshina	HG-U133B	230472_at	249	0.16	0.018268	0.25126	-0.61	0.41164	-0.1	0.90 [0.71 - 1.15]
IRX1	GSE9891	Ovarian cancer		Overall Survival	AOCS, RBH, WH, NKI-AVL (1992-2006)	Tothill	HG-U133_Plus_2	230472_at	278	0.39	0.093121	-	0.34	0.768861	-0.05	0.95 [0.70 - 1.30]
IRX1	GSE8841	Ovarian cancer		Overall Survival	Milan (1992-2003)	Marchini	G4100A	11570	81	0.53	0.008772	0.146226	-1.56	0.235178	-0.27	0.76 [0.49 - 1.19]
IRX1	GSE17260	Ovarian cancer		Overall Survival	Niigata (1997-2008)	Yoshihara	G4112A	A_23_P133457	110	0.16	0.13478	-	-0.56	0.330991	-0.41	0.67 [0.29 - 1.51]
IRX1	GSE17260	Ovarian cancer		Progression Free Survival	Niigata (1997-2008)	Yoshihara	G4112A	A_23_P133457	110	0.11	0.329896	-	0.41	0.708618	0.12	1.12 [0.61 - 2.06]

IRX2	GSE19615	Breast cancer		Distant Metastasis Free Survival	DF/HCC	Li	HG-U133_Plus_2	228404_at	115	0.47	0.142197	-	0.85	0.566339	0.45	1.56 [0.34 - 7.14]
IRX2	GSE19615	Breast cancer		Distant Metastasis Free Survival	DF/HCC	Li	HG-U133_Plus_2	228462_at	115	0.77	0.132026	-	-1.44	0.725263	-0.09	0.91 [0.54 - 1.54]
IRX2	GSE12276	Breast cancer		Relapse Free Survival	EMC	Bos	HG-U133_Plus_2	228404_at	204	0.82	0.011022	0.173688	-0.47	0.15974	-0.13	0.88 [0.73 - 1.05]
IRX2	GSE12276	Breast cancer		Relapse Free Survival	EMC	Bos	HG-U133_Plus_2	228462_at	204	0.79	0.007378	0.1281	-0.46	0.20471	-0.06	0.94 [0.86 - 1.03]
IRX2	GSE6532-GPL570	Breast cancer		Relapse Free Survival	GUYT	Loi	HG-U133_Plus_2	228462_at	87	0.4	0.164305	-	-0.52	0.984596	0	1.00 [0.83 - 1.20]
IRX2	GSE6532-GPL570	Breast cancer		Distant Metastasis Free Survival	GUYT	Loi	HG-U133_Plus_2	228462_at	87	0.4	0.164305	-	-0.52	0.984596	0	1.00 [0.83 - 1.20]
IRX2	GSE6532-GPL570	Breast cancer		Distant Metastasis Free Survival	GUYT	Loi	HG-U133_Plus_2	228404_at	87	0.3	0.091638	-	0.81	0.627799	0.27	1.31 [0.44 - 3.97]
IRX2	GSE6532-GPL570	Breast cancer		Relapse Free Survival	GUYT	Loi	HG-U133_Plus_2	228404_at	87	0.3	0.091638	-	0.81	0.627799	0.27	1.31 [0.44 - 3.97]
IRX2	GSE9195	Breast cancer		Distant Metastasis Free Survival	GUYT2	Loi	HG-U133_Plus_2	228404_at	77	0.16	0.15897	-	-0.94	0.631612	-0.49	0.61 [0.08 - 4.57]

IRX2	GSE9195	Breast cancer		Relapse Free Survival	GUYT2	Loi	HG-U133_Plus_2	228462_at	77	0.62	0.075808	-	-1.28	0.360399	-0.14	0.87 [0.64 - 1.17]
IRX2	GSE9195	Breast cancer		Distant Metastasis Free Survival	GUYT2	Loi	HG-U133_Plus_2	228462_at	77	0.12	0.040366	0.43017	-1.32	0.297943	-0.18	0.83 [0.59 - 1.18]
IRX2	GSE9195	Breast cancer		Relapse Free Survival	GUYT2	Loi	HG-U133_Plus_2	228404_at	77	0.52	0.310733	-	0.57	0.950115	-0.05	0.95 [0.17 - 5.17]
IRX2	GSE1378	Breast cancer		Relapse Free Survival	MGH (1987-2000)	Ma	Arcturus 22k	2675	60	0.88	0.096626	-	-1.54	0.19602	-0.87	0.42 [0.11 - 1.57]
IRX2	GSE1379	Breast cancer		Relapse Free Survival	MGH (1987-2000)	Ma	Arcturus 22k	2675	60	0.12	0.005834	0.106801	-1.24	0.076846	-1.65	0.19 [0.03 - 1.20]
IRX2	GSE1456-GPL97	Breast cancer		Overall Survival	Stockholm (1994-1996)	Pawitan	HG-U133B	228404_at	159	0.22	0.075941	-	-0.59	0.444107	-0.21	0.81 [0.48 - 1.39]
IRX2	GSE1456-GPL97	Breast cancer		Relapse Free Survival	Stockholm (1994-1996)	Pawitan	HG-U133B	228404_at	159	0.22	0.050757	-	-0.65	0.223239	-0.32	0.72 [0.43 - 1.22]
IRX2	GSE1456-GPL97	Breast cancer		Disease Specific Survival	Stockholm (1994-1996)	Pawitan	HG-U133B	228462_at	159	0.5	0.000606	0.016694	-1.44	0.040958	-0.33	0.72 [0.52 - 0.99]
IRX2	GSE1456-GPL97	Breast cancer		Disease Specific Survival	Stockholm (1994-1996)	Pawitan	HG-U133B	228404_at	159	0.22	0.025236	0.315055	-0.83	0.159772	-0.42	0.66 [0.37 - 1.18]
IRX2	GSE1456-GPL97	Breast cancer		Overall Survival	Stockholm (1994-1996)	Pawitan	HG-U133B	228462_at	159	0.48	0.025441	0.316806	-0.72	0.30984	-0.15	0.86 [0.65 - 1.15]
IRX2	GSE1456-GPL97	Breast cancer		Relapse Free Survival	Stockholm (1994-1996)	Pawitan	HG-U133B	228462_at	159	0.51	0.000392	0.011498	-1.22	0.03956	-0.29	0.75 [0.57 - 0.99]

IRX2	GSE3494-GPL97	Breast cancer		Disease Specific Survival	Uppsala (1987-1989)	Miller	HG-U133B	228404_at	236	0.21	0.036797	0.405318	0.88	0.706321	0.1	1.10 [0.66 - 1.83]
IRX2	GSE3494-GPL97	Breast cancer		Disease Specific Survival	Uppsala (1987-1989)	Miller	HG-U133B	228462_at	236	0.64	0.185002	-	-0.4	0.71633	-0.04	0.96 [0.76 - 1.21]
IRX2	GSE4922-GPL97	Breast cancer		Disease Free Survival	Uppsala (1987-1989)	Ivshina	HG-U133B	228404_at	249	0.62	0.00346	0.070645	-0.7	0.377806	-0.16	0.85 [0.59 - 1.22]
IRX2	GSE4922-GPL97	Breast cancer		Disease Free Survival	Uppsala (1987-1989)	Ivshina	HG-U133B	228462_at	249	0.47	0.034835	0.391114	-0.45	0.213279	-0.11	0.89 [0.74 - 1.07]
IRX2	GSE9891	Ovarian cancer		Overall Survival	AOCS, RBH, WH, NKI-AVL (1992-2006)	Tothill	HG-U133_Plus_2	228462_at	278	0.87	0.212857	-	0.33	0.478431	0.11	1.12 [0.82 - 1.52]
IRX2	GSE9891	Ovarian cancer		Overall Survival	AOCS, RBH, WH, NKI-AVL (1992-2006)	Tothill	HG-U133_Plus_2	228404_at	278	0.5	0.000459	0.01317	-0.67	0.09514	-0.94	0.39 [0.13 - 1.18]
IRX3	GSE19615	Breast cancer		Distant Metastasis Free Survival	DF/HCC	Li	HG-U133_Plus_2	229638_at	115	0.88	0.033251	0.379335	1.19	0.778352	0.15	1.16 [0.41 - 3.26]
IRX3	GSE12276	Breast cancer		Relapse Free Survival	EMC	Bos	HG-U133_Plus_2	229638_at	204	0.53	0.318561	-	0.14	0.750934	0.03	1.03 [0.85 - 1.26]
IRX3	GSE6532-GPL570	Breast cancer		Distant Metastasis Free Survival	GUYT	Loi	HG-U133_Plus_2	229638_at	87	0.68	0.018272	0.251303	-1.2	0.318844	-0.32	0.73 [0.39 - 1.36]

IRX3	GSE6532-GPL570	Breast cancer		Relapse Free Survival	GUYT	Loi	HG-U133_Plus_2	229638_at	87	0.68	0.018272	0.251303	-1.2	0.318844	-0.32	0.73 [0.39 - 1.36]
IRX3	GSE9195	Breast cancer		Distant Metastasis Free Survival	GUYT2	Loi	HG-U133_Plus_2	229638_at	77	0.87	0.206874	-	-15.24	0.074758	-0.58	0.56 [0.30 - 1.06]
IRX3	GSE9195	Breast cancer		Relapse Free Survival	GUYT2	Loi	HG-U133_Plus_2	229638_at	77	0.75	0.132463	-	-1.44	0.174847	-0.43	0.65 [0.35 - 1.21]
IRX3	GSE1378	Breast cancer		Relapse Free Survival	MGH (1987-2000)	Ma	Arcturus 22k	2599	60	0.78	0.150376	-	0.58	0.872214	-0.04	0.96 [0.56 - 1.65]
IRX3	GSE1379	Breast cancer		Relapse Free Survival	MGH (1987-2000)	Ma	Arcturus 22k	2599	60	0.52	0.003149	0.065502	1.16	0.367611	0.38	1.46 [0.64 - 3.30]
IRX3	GSE1456-GPL97	Breast cancer		Overall Survival	Stockholm (1994-1996)	Pawitan	HG-U133B	229638_at	159	0.9	0.187356	-	0.58	0.714153	0.11	1.12 [0.62 - 2.03]
IRX3	GSE1456-GPL97	Breast cancer		Relapse Free Survival	Stockholm (1994-1996)	Pawitan	HG-U133B	229638_at	159	0.89	0.007423	0.128702	1.02	0.516403	0.2	1.23 [0.66 - 2.27]
IRX3	GSE1456-GPL97	Breast cancer		Disease Specific Survival	Stockholm (1994-1996)	Pawitan	HG-U133B	229638_at	159	0.8	0.110314	-	0.63	0.919047	0.04	1.04 [0.52 - 2.08]
IRX3	GSE3494-GPL97	Breast cancer		Disease Specific Survival	Uppsala (1987-1989)	Miller	HG-U133B	229638_at	236	0.23	0.087078	-	0.64	0.985077	-0.01	0.99 [0.55 - 1.81]
IRX3	GSE4922-GPL97	Breast cancer		Disease Free Survival	Uppsala (1987-1989)	Ivshina	HG-U133B	229638_at	249	0.24	0.130085	-	0.41	0.979656	-0.01	0.99 [0.61 - 1.61]

IRX3	GSE9891	Ovarian cancer		Overall Survival	AOCS, RBH, WH, NKI-AVL (1992-2006)	Tothill	HG-U133_Plus_2	229638_at	278	0.19	0.048416	0.482031	0.54	0.099042	0.1	1.10 [0.98 - 1.23]
IRX3	GSE17260	Ovarian cancer		Progression Free Survival	Niigata (1997-2008)	Yoshihara	G4112A	A_24_P207195	110	0.31	0.005459	0.1014	-0.65	0.076306	-0.36	0.70 [0.47 - 1.04]
IRX3	GSE17260	Ovarian cancer		Overall Survival	Niigata (1997-2008)	Yoshihara	G4112A	A_24_P207195	110	0.58	0.001099	0.027577	-1.12	0.007812	-0.68	0.51 [0.31 - 0.84]
IRX3	GSE17260	Ovarian cancer		Progression Free Survival	Niigata (1997-2008)	Yoshihara	G4112A	A_23_P152235	110	0.68	0.079035	-	-0.45	0.580983	-0.02	0.98 [0.90 - 1.06]
IRX3	GSE17260	Ovarian cancer		Overall Survival	Niigata (1997-2008)	Yoshihara	G4112A	A_23_P152235	110	0.42	0.159621	-	-0.41	0.650009	-0.02	0.98 [0.88 - 1.08]
IRX4	GSE19615	Breast cancer		Distant Metastasis Free Survival	DF/HCC	Li	HG-U133_Plus_2	220225_at	115	0.6	0.011632	0.180797	1.38	0.009716	0.77	2.16 [1.20 - 3.87]
IRX4	GSE12276	Breast cancer		Relapse Free Survival	EMC	Bos	HG-U133_Plus_2	220225_at	204	0.5	0.008105	0.137677	0.38	0.372808	0.05	1.06 [0.94 - 1.19]
IRX4	GSE6532-GPL570	Breast cancer		Relapse Free Survival	GUYT	Loi	HG-U133_Plus_2	220225_at	87	0.69	0.190483	-	0.5	0.754924	0.08	1.08 [0.67 - 1.74]
IRX4	GSE6532-GPL570	Breast cancer		Distant Metastasis Free Survival	GUYT	Loi	HG-U133_Plus_2	220225_at	87	0.69	0.190483	-	0.5	0.754924	0.08	1.08 [0.67 - 1.74]

IRX4	GSE9195	Breast cancer		Distant Metastasis Free Survival	GUYT2	Loi	HG-U133_Plus_2	220225_at	77	0.12	0.000635	0.017369	-1.92	0.359896	-0.45	0.64 [0.24 - 1.67]
IRX4	GSE9195	Breast cancer		Relapse Free Survival	GUYT2	Loi	HG-U133_Plus_2	220225_at	77	0.12	0.008566	0.14361	-1.46	0.748915	-0.12	0.89 [0.42 - 1.87]
IRX4	GSE12093	Breast cancer		Distant Metastasis Free Survival	IO, NCI, TUM, CCF (1992-2000)	Zhang	HG-U133A	220225_at	136	0.76	0.038328	0.416133	-1.85	0.335174	-0.21	0.81 [0.53 - 1.24]
IRX4	GSE11121	Breast cancer		Distant Metastasis Free Survival	Mainz (1988-1998)	Schmidt	HG-U133A	220225_at	200	0.85	0.001722	0.040053	0.99	0.135091	0.25	1.29 [0.92 - 1.79]
IRX4	GSE1378	Breast cancer		Relapse Free Survival	MGH (1987-2000)	Ma	Arcturus 22k	18420	60	0.82	0.092257	-	0.72	0.356362	0.07	1.07 [0.93 - 1.24]
IRX4	GSE1379	Breast cancer		Relapse Free Survival	MGH (1987-2000)	Ma	Arcturus 22k	18420	60	0.83	0.038441	0.416923	0.88	0.552981	0.06	1.06 [0.88 - 1.28]
IRX4	GSE9893	Breast cancer		Overall Survival	Montpellier, Bordeaux, Turin (1989-2001)	Chanrion	MLRG Human 21K V12.0	9581	155	0.12	0.043707	0.452358	1.14	0.311116	0.23	1.25 [0.81 - 1.94]
IRX4	GSE2034	Breast cancer		Distant Metastasis Free Survival	Rotterdam (1980-1995)	Wang	HG-U133A	220225_at	286	0.1	0.021231	0.279451	-0.63	0.241804	-0.1	0.90 [0.76 - 1.07]
IRX4	GSE1456-GPL96	Breast cancer		Disease Specific Survival	Stockholm (1994-1996)	Pawitan	HG-U133A	220225_at	159	0.3	0.030598	0.358968	-0.79	0.341373	-0.19	0.83 [0.57 - 1.22]

IRX4	GSE1456-GPL96	Breast cancer		Overall Survival	Stockholm (1994-1996)	Pawitan	HG-U133A	220225_at	159	0.3	0.010616	0.168875	-0.79	0.388428	-0.14	0.87 [0.62 - 1.20]
IRX4	GSE1456-GPL96	Breast cancer		Relapse Free Survival	Stockholm (1994-1996)	Pawitan	HG-U133A	220225_at	159	0.14	0.001848	0.042453	-1.05	0.025757	-0.37	0.69 [0.50 - 0.96]
IRX4	GSE7378	Breast cancer		Disease Free Survival	UCSF	Zhou	U133AAofAv2	220225_at	54	0.11	0.015455	0.222787	-1.8	0.048578	0.75	2.13 [1.00 - 4.50]
IRX4	E-TABM-158	Breast cancer		Overall Survival	UCSF, CPMC (1989-1997)	Chin	HG-U133A	220225_at	117	0.56	0.096573	-	-0.56	0.941379	0.01	1.01 [0.69 - 1.49]
IRX4	E-TABM-158	Breast cancer		Disease Specific Survival	UCSF, CPMC (1989-1997)	Chin	HG-U133A	220225_at	117	0.56	0.024958	0.312668	-0.94	0.415448	-0.22	0.80 [0.48 - 1.36]
IRX4	E-TABM-158	Breast cancer		Distant Metastasis Free Survival	UCSF, CPMC (1989-1997)	Chin	HG-U133A	220225_at	117	0.56	0.105808	-	-0.67	0.552756	-0.16	0.85 [0.51 - 1.44]
IRX4	E-TABM-158	Breast cancer		Relapse Free Survival	UCSF, CPMC (1989-1997)	Chin	HG-U133A	220225_at	117	0.56	0.096573	-	-0.56	0.941379	0.01	1.01 [0.69 - 1.49]
IRX4	GSE3494-GPL96	Breast cancer		Disease Specific Survival	Uppsala (1987-1989)	Miller	HG-U133A	220225_at	236	0.42	0.003298	0.067974	-0.79	0.080478	-0.2	0.82 [0.65 - 1.02]
IRX4	GSE4922-GPL96	Breast cancer		Disease Free Survival	Uppsala (1987-1989)	Ivshina	HG-U133A	220225_at	249	0.42	0.112397	-	-0.33	0.469437	-0.07	0.93 [0.78 - 1.12]
IRX4	GSE2990	Breast cancer		Relapse Free Survival	Uppsala, Oxford	Sotiriou	HG-U133A	220225_at	125	0.88	0.039684	0.425518	0.79	0.119251	0.41	1.50 [0.90 - 2.51]

IRX4	GSE2990	Breast cancer		Distant Metastasis Free Survival	Uppsala, Oxford	Sotiriou	HG-U133A	220225_at	54	0.72	0.040903	0.433807	-1.9	0.749585	-0.08	0.93 [0.58 - 1.47]
IRX4	GSE2990	Breast cancer		Relapse Free Survival	Uppsala, Oxford	Sotiriou	HG-U133A	220225_at	62	0.79	0.095315	-	-1.2	0.669953	-0.08	0.92 [0.63 - 1.35]
IRX4	GSE2990	Breast cancer		Distant Metastasis Free Survival	Uppsala, Oxford	Sotiriou	HG-U133A	220225_at	125	0.9	0.094961	-	0.81	0.24766	0.38	1.46 [0.77 - 2.80]
IRX4	GSE7390	Breast cancer		Relapse Free Survival	Uppsala, Oxford, Stockholm, IGR, GUYT, CRH (1980-1998)	Desmedt	HG-U133A	220225_at	198	0.27	0.033122	0.378369	0.55	0.215937	0.08	1.08 [0.96 - 1.22]
IRX4	GSE7390	Breast cancer		Overall Survival	Uppsala, Oxford, Stockholm, IGR, GUYT, CRH (1980-1998)	Desmedt	HG-U133A	220225_at	198	0.27	0.045389	0.463164	0.71	0.310687	0.08	1.08 [0.93 - 1.27]
IRX4	GSE7390	Breast cancer		Distant Metastasis Free Survival	Uppsala, Oxford, Stockholm, IGR, GUYT, CRH (1980-1998)	Desmedt	HG-U133A	220225_at	198	0.27	0.038474	0.417149	0.68	0.257478	0.09	1.09 [0.94 - 1.26]

IRX4	GSE9891	Ovarian cancer		Overall Survival	AOCS, RBH, WH, NKI-AVL (1992-2006)	Tothill	HG-U133_Plus_2	220225_at	278	0.39	0.058536	-	0.38	0.61503	0.28	1.32 [0.45 - 3.87]
IRX4	DUKE-OC	Ovarian cancer		Overall Survival	Duke	Bild	HG-U133A	220225_at	133	0.89	0.000727	0.019471	1	0.005041	2.6	13.48 [2.19 - 83.05]
IRX4	GSE8841	Ovarian cancer		Overall Survival	Milan (1992-2003)	Marchini	G4100A	4889	81	0.88	0.003587	0.07271	1.67	0.539934	0.31	1.36 [0.51 - 3.68]
IRX4	GSE26712	Ovarian cancer		Overall Survival	MSKCC (1990-2003)	Bonome	HG-U133_Plus_2	220225_at	185	0.69	0.075143	-	-0.35	0.960597	-0.02	0.98 [0.51 - 1.90]
IRX4	GSE26712	Ovarian cancer		Disease Free Survival	MSKCC (1990-2003)	Bonome	HG-U133_Plus_2	220225_at	185	0.69	0.014205	0.209518	-0.45	0.646269	-0.15	0.86 [0.46 - 1.61]
IRX4	GSE17260	Ovarian cancer		Overall Survival	Niigata (1997-2008)	Yoshihara	G4112A	A_24_P20795	110	0.39	0.09374	-	-0.49	0.22421	-0.41	0.66 [0.34 - 1.29]
IRX4	GSE17260	Ovarian cancer		Progression Free Survival	Niigata (1997-2008)	Yoshihara	G4112A	A_24_P20795	110	0.15	0.322133	-	0.33	0.836029	0.05	1.05 [0.64 - 1.72]
IRX4	GSE17260	Ovarian cancer		Overall Survival	Niigata (1997-2008)	Yoshihara	G4112A	A_23_P110837	110	0.87	0.310607	-	-0.61	0.811323	-0.06	0.94 [0.58 - 1.53]
IRX4	GSE17260	Ovarian cancer		Progression Free Survival	Niigata (1997-2008)	Yoshihara	G4112A	A_23_P110837	110	0.13	0.164112	-	0.52	0.873206	-0.03	0.97 [0.69 - 1.36]

IRX4	GSE14764	Ovarian cancer		Overall Survival	TOC	Denkert	HG-U133A	220225_at	80	0.55	0.067203	-	-0.91	0.206317	-0.26	0.77 [0.52 - 1.15]
IRX4	GSE16560	Prostate cancer		Overall Survival	Sweden (1977-1999)	Sboner	6K DASL	DAP3_0087	281	0.11	0.221023	-	0.29	0.936012	0.01	1.01 [0.88 - 1.15]
IRX5	GSE19615	Breast cancer		Distant Metastasis Free Survival	DF/HCC	Li	HG-U133_Plus_2	210239_at	115	0.1	0.148228	-	-0.91	0.783627	-0.11	0.90 [0.42 - 1.92]
IRX5	GSE3143	Breast cancer		Overall Survival	Duke	Bild	HG-U95A	41348_at	158	0.14	0.017019	0.238848	-0.83	0.239931	-0.17	0.84 [0.63 - 1.12]
IRX5	GSE3143	Breast cancer		Overall Survival	Duke	Bild	HG-U95A	41347_at	158	0.11	0.003042	0.063697	-1.07	0.026834	-0.67	0.51 [0.28 - 0.93]
IRX5	GSE7849	Breast cancer		Disease Free Survival	Duke (1990-2001)	Anders	HG-U95A	41347_at	76	0.11	0.011789	0.182617	-1.38	0.068149	-1	0.37 [0.13 - 1.08]
IRX5	GSE7849	Breast cancer		Disease Free Survival	Duke (1990-2001)	Anders	HG-U95A	41348_at	76	0.84	0.042405	0.443835	1.07	0.137205	0.54	1.72 [0.84 - 3.52]
IRX5	GSE12276	Breast cancer		Relapse Free Survival	EMC	Bos	HG-U133_Plus_2	210239_at	204	0.25	0.032001	0.369848	-0.35	0.437181	-0.07	0.94 [0.79 - 1.11]
IRX5	GSE6532-GPL570	Breast cancer		Relapse Free Survival	GUYT	Loi	HG-U133_Plus_2	210239_at	87	0.43	0.010914	0.172415	-0.96	0.244328	-0.31	0.73 [0.43 - 1.24]
IRX5	GSE6532-GPL570	Breast cancer		Distant Metastasis Free Survival	GUYT	Loi	HG-U133_Plus_2	210239_at	87	0.43	0.010914	0.172415	-0.96	0.244328	-0.31	0.73 [0.43 - 1.24]

IRX5	GSE9195	Breast cancer		Relapse Free Survival	GUYT2	Loi	HG-U133_Plus_2	210239_at	77	0.47	0.189608	-	-0.73	0.394512	-0.25	0.78 [0.43 - 1.39]
IRX5	GSE9195	Breast cancer		Distant Metastasis Free Survival	GUYT2	Loi	HG-U133_Plus_2	210239_at	77	0.1	0.242374	-	-0.89	0.319607	-0.33	0.72 [0.37 - 1.38]
IRX5	GSE12093	Breast cancer		Distant Metastasis Free Survival	IO, NCI, TUM, CCF (1992-2000)	Zhang	HG-U133A	210239_at	136	0.22	0.059242	-	1.72	0.607965	0.2	1.22 [0.57 - 2.58]
IRX5	GSE11121	Breast cancer		Distant Metastasis Free Survival	Mainz (1988-1998)	Schmidt	HG-U133A	210239_at	200	0.13	0.020365	0.271385	-0.78	0.167087	-0.41	0.66 [0.37 - 1.19]
IRX5	GSE1378	Breast cancer		Relapse Free Survival	MGH (1987-2000)	Ma	Arcturus 22k	14047	60	0.3	0.181639	-	0.61	0.595095	-0.14	0.87 [0.51 - 1.47]
IRX5	GSE1379	Breast cancer		Relapse Free Survival	MGH (1987-2000)	Ma	Arcturus 22k	14047	60	0.17	0.22292	-	0.73	0.707689	-0.09	0.92 [0.59 - 1.43]
IRX5	GSE9893	Breast cancer		Overall Survival	Montpellier, Bordeaux, Turin (1989-2001)	Chanrion	MLRG Human 21K V12.0	16577	155	0.85	0.000169	0.005559	1.07	0.003503	0.29	1.33 [1.10 - 1.62]
IRX5	GSE2034	Breast cancer		Distant Metastasis Free Survival	Rotterdam (1980-1995)	Wang	HG-U133A	210239_at	286	0.76	0.085855	-	0.36	0.77218	-0.04	0.96 [0.71 - 1.29]
IRX5	GSE1456-GPL96	Breast cancer		Overall Survival	Stockholm (1994-1996)	Pawitan	HG-U133A	210239_at	159	0.57	0.003303	0.068063	0.93	0.041965	0.55	1.73 [1.02 - 2.94]

IRX5	GSE1456-GPL96	Breast cancer		Disease Specific Survival	Stockholm (1994-1996)	Pawitan	HG-U133A	210239_at	159	0.3	0.033972	0.384727	1.09	0.146012	0.46	1.58 [0.85 - 2.92]
IRX5	GSE1456-GPL96	Breast cancer		Relapse Free Survival	Stockholm (1994-1996)	Pawitan	HG-U133A	210239_at	159	0.76	0.006081	0.110307	0.86	0.034729	0.58	1.78 [1.04 - 3.03]
IRX5	GSE7378	Breast cancer		Disease Free Survival	UCSF	Zhou	U133A AofAv2	210239_at	54	0.65	0.014006	0.207364	1.77	0.057801	0.88	2.42 [0.97 - 6.01]
IRX5	E-TABM-158	Breast cancer		Disease Specific Survival	UCSF, CPMC (1989-1997)	Chin	HG-U133A	210239_at	117	0.1	0.041025	0.434631	15.23	0.294678	0.2	1.22 [0.84 - 1.76]
IRX5	E-TABM-158	Breast cancer		Overall Survival	UCSF, CPMC (1989-1997)	Chin	HG-U133A	210239_at	117	0.1	0.048992	0.485541	1.76	0.3415	0.15	1.16 [0.85 - 1.57]
IRX5	E-TABM-158	Breast cancer		Relapse Free Survival	UCSF, CPMC (1989-1997)	Chin	HG-U133A	210239_at	117	0.1	0.048992	0.485541	1.76	0.3415	0.15	1.16 [0.85 - 1.57]
IRX5	E-TABM-158	Breast cancer		Distant Metastasis Free Survival	UCSF, CPMC (1989-1997)	Chin	HG-U133A	210239_at	117	0.38	0.15085	-	0.63	0.634948	0.09	1.09 [0.76 - 1.58]
IRX5	GSE3494-GPL96	Breast cancer		Disease Specific Survival	Uppsala (1987-1989)	Miller	HG-U133A	210239_at	236	0.56	0.136275	-	-0.42	0.883762	-0.04	0.97 [0.60 - 1.54]
IRX5	GSE4922-GPL96	Breast cancer		Disease Free Survival	Uppsala (1987-1989)	Ivshina	HG-U133A	210239_at	249	0.31	0.143669	-	0.36	0.580827	0.11	1.11 [0.76 - 1.61]
IRX5	GSE2990	Breast cancer		Distant Metastasis Free Survival	Uppsala, Oxford	Sotiriou	HG-U133A	210239_at	54	0.65	0.070893	-	-1.32	0.5352	-0.21	0.81 [0.41 - 1.58]

IRX5	GSE2990	Breast cancer		Relapse Free Survival	Uppsala, Oxford	Sotiriou	HG-U133A	210239_at	125	0.14	0.032769	0.375697	1.42	0.185573	0.25	1.28 [0.89 - 1.86]
IRX5	GSE2990	Breast cancer		Relapse Free Survival	Uppsala, Oxford	Sotiriou	HG-U133A	210239_at	62	0.66	0.11561	-	-0.88	0.477707	-0.19	0.82 [0.48 - 1.40]
IRX5	GSE2990	Breast cancer		Distant Metastasis Free Survival	Uppsala, Oxford	Sotiriou	HG-U133A	210239_at	125	0.35	0.025881	0.320549	1.05	0.11398	0.4	1.49 [0.91 - 2.43]
IRX5	GSE7390	Breast cancer		Relapse Free Survival	Uppsala, Oxford, Stockholm, IGR, GUYT, CRH (1980-1998)	Desmedt	HG-U133A	210239_at	198	0.55	0.005422	0.100855	0.59	0.028844	0.24	1.27 [1.03 - 1.58]
IRX5	GSE7390	Breast cancer		Overall Survival	Uppsala, Oxford, Stockholm, IGR, GUYT, CRH (1980-1998)	Desmedt	HG-U133A	210239_at	198	0.48	0.094077	-	0.45	0.416185	0.11	1.12 [0.86 - 1.45]
IRX5	GSE7390	Breast cancer		Distant Metastasis Free Survival	Uppsala, Oxford, Stockholm, IGR, GUYT, CRH (1980-1998)	Desmedt	HG-U133A	210239_at	198	0.47	0.099581	-	0.43	0.43306	0.1	1.10 [0.86 - 1.41]

IRX5	GSE9891	Ovarian cancer		Overall Survival	AOCS, RBH, WH, NKI-AVL (1992-2006)	Tothill	HG-U133_Plus_2	210239_at	278	0.86	0.002968	0.06244	0.66	0.051427	0.15	1.16 [1.00 - 1.34]
IRX5	DUKE-OC	Ovarian cancer		Overall Survival	Duke	Bild	HG-U133A	210239_at	133	0.54	0.070099	-	0.43	0.983172	0	1.00 [0.76 - 1.32]
IRX5	GSE8841	Ovarian cancer		Overall Survival	Milan (1992-2003)	Marchini	G4100A	5796	81	0.25	0.209099	-	-0.67	0.422782	-0.27	0.76 [0.40 - 1.48]
IRX5	GSE26712	Ovarian cancer		Disease Free Survival	MSKCC (1990-2003)	Bonome	HG-U133_Plus_2	210239_at	185	0.44	0.071026	-	-0.29	0.474277	-0.14	0.87 [0.60 - 1.27]
IRX5	GSE26712	Ovarian cancer		Overall Survival	MSKCC (1990-2003)	Bonome	HG-U133_Plus_2	210239_at	185	0.43	0.030037	0.354551	-0.38	0.211013	-0.27	0.76 [0.50 - 1.16]
IRX5	GSE17260	Ovarian cancer		Overall Survival	Niigata (1997-2008)	Yoshihara	G4112A	A_23_P9779	110	0.59	0.020615	0.273726	0.68	0.310112	0.07	1.07 [0.94 - 1.23]
IRX5	GSE17260	Ovarian cancer		Progression Free Survival	Niigata (1997-2008)	Yoshihara	G4112A	A_24_P48057	110	0.37	0.087379	-	0.41	0.98777	0	1.00 [0.84 - 1.18]
IRX5	GSE17260	Ovarian cancer		Overall Survival	Niigata (1997-2008)	Yoshihara	G4112A	A_24_P48057	110	0.83	0.192694	-	-0.61	0.918472	-0.01	0.99 [0.80 - 1.23]
IRX5	GSE17260	Ovarian cancer		Progression Free Survival	Niigata (1997-2008)	Yoshihara	G4112A	A_23_P9779	110	0.85	0.063086	-	0.54	0.510069	0.04	1.04 [0.93 - 1.15]

IRX5	GSE14764	Ovarian cancer		Overall Survival	TOC	Denkert	HG-U133A	210239_at	80	0.8	0.001265	0.031017	1.41	0.080329	0.22	1.25 [0.97 - 1.60]
IRX5	GSE16560	Prostate cancer		Overall Survival	Sweden (1977-1999)	Sboner	6K DASL	DAP4_2896	281	0.12	0.340105	-	-0.2	0.842549	-0.02	0.98 [0.83 - 1.16]
IRX6	GSE1378	Breast cancer		Relapse Free Survival	MGH (1987-2000)	Ma	Arcturus 22k	14245	60	0.57	0.147006	-	-0.57	0.234638	-1.36	0.26 [0.03 - 2.42]
IRX6	GSE1379	Breast cancer		Relapse Free Survival	MGH (1987-2000)	Ma	Arcturus 22k	14245	60	0.75	0.046992	0.473246	-1.15	0.462497	-0.86	0.43 [0.04 - 4.16]
IRX6	GSE17260	Ovarian cancer		Progression Free Survival	Niigata (1997-2008)	Yoshihara	G4112A	A_32_P177539	110	0.13	0.176075	-	0.5	0.784469	0.07	1.08 [0.64 - 1.82]
IRX6	GSE17260	Ovarian cancer		Overall Survival	Niigata (1997-2008)	Yoshihara	G4112A	A_32_P177539	110	0.21	0.269718	-	-0.38	0.5725	-0.21	0.81 [0.40 - 1.67]
IRX6	GSE17260	Ovarian cancer		Progression Free Survival	Niigata (1997-2008)	Yoshihara	G4112A	A_23_P73348	110	0.88	0.086742	-	-0.71	0.710819	-0.07	0.93 [0.64 - 1.35]
IRX6	GSE17260	Ovarian cancer		Overall Survival	Niigata (1997-2008)	Yoshihara	G4112A	A_23_P73348	110	0.87	0.053079	-	-1.11	0.598807	-0.14	0.87 [0.53 - 1.44]
IRX6	GSE17260	Ovarian cancer		Progression Free Survival	Niigata (1997-2008)	Yoshihara	G4112A	A_32_P519101	110	0.72	0.090474	-	0.4	0.730549	0.07	1.07 [0.72 - 1.60]

IRX6	GSE17260	Ovarian cancer		Overall Survival	Niigata (1997-2008)	Yoshihara	G4112A	A_32_P519101	110	0.76	0.086714	-	0.53	0.795203	0.07	1.07 [0.64 - 1.80]

Supplementary Table S4: Correlation coefficient values of IRXs with drugs in hormone sensitive cancers

Gene	Drug	Correlation	P_Value	Adj_P_Value
IRX1	Thiotepa	-0.079558448	0.786887577	0.96336506
IRX1	Cyclophosphamide	0.414869907	0.306767817	0.93163245
IRX1	Megestrol acetate	-0.682407094	0.007166614	0.150498887
IRX1	Cisplatin	0.165564528	0.571627106	0.96336506
IRX1	Doxorubicin	-0.077263446	0.792911555	0.96336506
IRX1	Tamoxifen	0.501660215	0.067594398	0.686239837
IRX1	Carboplatin	-0.157427938	0.590926343	0.96336506
IRX1	Docetaxel	0.263157895	0.668845465	0.96336506
IRX1	Fulvestrant	-0.105074257	0.720725872	0.96336506
IRX1	Everolimus	-0.289851307	0.314797444	0.93163245
IRX1	Olaparib	-0.001782761	0.995612756	0.995612756
IRX1	Ixabepilone	-0.007042428	0.98267017	0.990531532
IRX1	Abiraterone	-0.487755396	0.07684621	0.691615893
IRX1	Olaparib	-0.264231703	0.361314741	0.93163245
IRX1	Enzalutamide	-0.356984479	0.210215197	0.913348787
IRX1	Lenvatinib	-0.194476207	0.505265789	0.96336506
IRX1	Fluorouracil	-0.028824834	0.922078221	0.981448491
IRX1	Melphalan	-0.174586595	0.550538393	0.96336506
IRX1	Palbociclib	0.190056293	0.515176662	0.96336506
IRX1	Paclitaxel	0.187639798	0.520631995	0.96336506
IRX1	Docetaxel	0.14228912	0.642856129	0.96336506
IRX2	Thiotepa	0.006629871	0.982054048	0.990531532
IRX2	Cyclophosphamide	0.082973981	0.845136372	0.96336506
IRX2	Megestrol acetate	-0.287208373	0.319430152	0.93163245
IRX2	Cisplatin	0.116998933	0.690388522	0.96336506
IRX2	Doxorubicin	0.364241961	0.200425414	0.901914364
IRX2	Tamoxifen	0.800004396	0.00059134	0.037254434
IRX2	Carboplatin	-0.308203991	0.283696402	0.93163245
IRX2	Docetaxel	-0.153896753	0.804828817	0.96336506
IRX2	Fulvestrant	0.254390306	0.380124643	0.93163245
IRX2	Everolimus	0.117320767	0.689575481	0.96336506
IRX2	Olaparib	0.412210211	0.183004366	0.901914364
IRX2	Ixabepilone	0.204230416	0.524324327	0.96336506
IRX2	Abiraterone	-0.501118557	0.067939139	0.686239837
IRX2	Olaparib	0.241255033	0.406023587	0.93163245
IRX2	Enzalutamide	0.11308204	0.700308316	0.96336506
IRX2	Lenvatinib	-0.114917758	0.695653597	0.96336506
IRX2	Fluorouracil	0.011086475	0.969994714	0.990531532
IRX2	Melphalan	-0.103867974	0.723816964	0.96336506

IRX2	Palbociclib	0.212155862	0.466517073	0.96336506
IRX2	Paclitaxel	0.417222609	0.137744827	0.867792408
IRX2	Docetaxel	0.156518032	0.609605082	0.96336506
IRX3	Thiotepa	-0.028602878	0.92267659	0.981448491
IRX3	Cyclophosphamide	0.412393049	0.309958736	0.93163245
IRX3	Megestrol acetate	-0.173853424	0.55223964	0.96336506
IRX3	Cisplatin	-0.125274725	0.670604371	0.96336506
IRX3	Doxorubicin	0.323076923	0.259694007	0.93163245
IRX3	Tamoxifen	0.4224425	0.132385486	0.867792408
IRX3	Carboplatin	-0.169979582	0.561265469	0.96336506
IRX3	Docetaxel	-0.051298918	0.934712848	0.981448491
IRX3	Fulvestrant	0.514798069	0.059609976	0.686239837
IRX3	Everolimus	0.141996938	0.628219283	0.96336506
IRX3	Olaparib	0.43779173	0.154625613	0.885583055
IRX3	Ixabepilone	0.328671329	0.297385078	0.93163245
IRX3	Abiraterone	0.068738846	0.815378891	0.96336506
IRX3	Olaparib	0.258492591	0.372221409	0.93163245
IRX3	Enzalutamide	0.125829041	0.668196445	0.96336506
IRX3	Lenvatinib	0.409241172	0.14621677	0.87730062
IRX3	Fluorouracil	0.072848392	0.804530283	0.96336506
IRX3	Melphalan	-0.074807526	0.799369821	0.96336506
IRX3	Palbociclib	-0.063806419	0.82844034	0.96336506
IRX3	Paclitaxel	0.459340659	0.100761748	0.793498769
IRX3	Docetaxel	0.342442468	0.252066825	0.93163245
IRX4	Thiotepa	0.089207048	0.761686191	0.96336506
IRX4	Cyclophosphamide	0.24743583	0.554646423	0.96336506
IRX4	Megestrol acetate	-0.271372464	0.347990768	0.93163245
IRX4	Cisplatin	0.063806419	0.82844034	0.96336506
IRX4	Doxorubicin	0.37403763	0.187677554	0.901914364
IRX4	Tamoxifen	0.732378855	0.002894897	0.091370688
IRX4	Carboplatin	-0.349173189	0.221081707	0.928543169
IRX4	Docetaxel	-0.461690258	0.433766156	0.93163245
IRX4	Fulvestrant	0.093702676	0.750016979	0.96336506
IRX4	Everolimus	-0.137567648	0.639082933	0.96336506
IRX4	Olaparib	0.345855561	0.270815488	0.93163245
IRX4	Ixabepilone	0.293706294	0.354332534	0.93163245
IRX4	Abiraterone	-0.213102991	0.464482662	0.96336506
IRX4	Olaparib	0.171754724	0.557121802	0.96336506
IRX4	Enzalutamide	0.03535931	0.904480402	0.981448491
IRX4	Lenvatinib	0.114537445	0.696617123	0.96336506
IRX4	Fluorouracil	0.148067112	0.613444328	0.96336506
IRX4	Melphalan	-0.02753304	0.925561284	0.981448491

IRX4	Palbociclib	0.089207048	0.761686191	0.96336506
IRX4	Paclitaxel	0.539054232	0.046687198	0.686239837
IRX4	Docetaxel	-0.086436957	0.77888265	0.96336506
IRX5	Thiotepa	0.226622799	0.43591643	0.93163245
IRX5	Cyclophosphamide	-0.577350269	0.133974596	0.867792408
IRX5	Megestrol acetate	0.22646696	0.436240592	0.93163245
IRX5	Cisplatin	-0.063736264	0.831924956	0.96336506
IRX5	Doxorubicin	0.705494505	0.006405395	0.150498887
IRX5	Tamoxifen	0.310231211	0.280376239	0.93163245
IRX5	Carboplatin	-0.240620447	0.407297446	0.93163245
IRX5	Docetaxel	-0.102597835	0.869597921	0.978297661
IRX5	Fulvestrant	0.732279606	0.002900657	0.091370688
IRX5	Everolimus	0.377895077	0.182803691	0.901914364
IRX5	Olaparib	0.320335413	0.310048966	0.93163245
IRX5	Ixabepilone	0.286713287	0.366408456	0.93163245
IRX5	Abiraterone	-0.090912667	0.757253266	0.96336506
IRX5	Olaparib	0.466659191	0.092538781	0.777325757
IRX5	Enzalutamide	0.496693583	0.070802523	0.686239837
IRX5	Lenvatinib	0.204620586	0.482854334	0.96336506
IRX5	Fluorouracil	0.249450555	0.389758912	0.93163245
IRX5	Melphalan	0.198019922	0.497383236	0.96336506
IRX5	Palbociclib	0.147414831	0.615025596	0.96336506
IRX5	Paclitaxel	0.261538462	0.36558891	0.93163245
IRX5	Docetaxel	0.237728656	0.434161943	0.93163245
IRX6	Thiotepa	0.162016759	0.580010041	0.96336506
IRX6	Cyclophosphamide	-0.35149982	0.39321139	0.93163245
IRX6	Megestrol acetate	-0.423456985	0.131360412	0.867792408
IRX6	Cisplatin	0.289960863	0.314606239	0.93163245
IRX6	Doxorubicin	0.366384656	0.197591331	0.901914364
IRX6	Tamoxifen	0.526554468	0.053061143	0.686239837
IRX6	Carboplatin	-0.3002747	0.296903614	0.93163245
IRX6	Docetaxel	0.057353933	0.927014759	0.981448491
IRX6	Fulvestrant	0.275919534	0.339649947	0.93163245
IRX6	Everolimus	0.025765647	0.930328701	0.981448491
IRX6	Olaparib	-0.007246377	0.982168368	0.990531532
IRX6	Ixabepilone	-0.014502276	0.964320812	0.990531532
IRX6	Abiraterone	-0.818669879	0.000343	0.037254434
IRX6	Olaparib	0.156749271	0.592547752	0.96336506
IRX6	Enzalutamide	0.056442613	0.848016255	0.96336506
IRX6	Lenvatinib	-0.389290269	0.168884768	0.901914364
IRX6	Fluorouracil	0.065473431	0.824021185	0.96336506
IRX6	Melphalan	0.042754423	0.884614272	0.981448491

IRX6	Palbociclib	0.542306098	0.045124876	0.686239837
IRX6	Paclitaxel	0.056193966	0.848678744	0.96336506
IRX6	Docetaxel	0.107714816	0.726143438	0.96336506
